# Phosphate Sink Containing Two-Component Signaling Systems as Tunable Threshold Devices

**DOI:** 10.1371/journal.pcbi.1003890

**Published:** 2014-10-30

**Authors:** Munia Amin, Varun B. Kothamachu, Elisenda Feliu, Birgit E. Scharf, Steven L. Porter, Orkun S. Soyer

**Affiliations:** 1 Biosciences, College of Life and Environmental Sciences, University of Exeter, Exeter, United Kingdom; 2 Systems Biology Program, College of Engineering, Mathematics and Physical Sciences, University of Exeter, Exeter, United Kingdom; 3 Department of Mathematical Sciences, University of Copenhagen, Copenhagen, Denmark; 4 Department of Biological Sciences, Virginia Tech, Blacksburg, Virginia, United States of America; 5 School of Life Sciences, University of Warwick, Coventry, United Kingdom; Johns Hopkins University, United States of America

## Abstract

Synthetic biology aims to design *de novo* biological systems and reengineer existing ones. These efforts have mostly focused on transcriptional circuits, with reengineering of signaling circuits hampered by limited understanding of their systems dynamics and experimental challenges. Bacterial two-component signaling systems offer a rich diversity of sensory systems that are built around a core phosphotransfer reaction between histidine kinases and their output response regulator proteins, and thus are a good target for reengineering through synthetic biology. Here, we explore the signal-response relationship arising from a specific motif found in two-component signaling. In this motif, a single histidine kinase (HK) phosphotransfers reversibly to two separate output response regulator (RR) proteins. We show that, under the experimentally observed parameters from bacteria and yeast, this motif not only allows rapid signal termination, whereby one of the RRs acts as a phosphate sink towards the other RR (i.e. the output RR), but also implements a sigmoidal signal-response relationship. We identify two mathematical conditions on system parameters that are necessary for sigmoidal signal-response relationships and define key parameters that control threshold levels and sensitivity of the signal-response curve. We confirm these findings experimentally, by *in vitro* reconstitution of the one HK-two RR motif found in the *Sinorhizobium meliloti* chemotaxis pathway and measuring the resulting signal-response curve. We find that the level of sigmoidality in this system can be experimentally controlled by the presence of the sink RR, and also through an auxiliary protein that is shown to bind to the HK (yielding Hill coefficients of above 7). These findings show that the one HK-two RR motif allows bacteria and yeast to implement tunable switch-like signal processing and provides an ideal basis for developing threshold devices for synthetic biology applications.

## Author Summary

Two-component signaling systems are found in bacteria, fungi and plants. Their modular structures make them ideal targets for *de novo* engineering through synthetic biology. Here, we explore the signal-response relationship arising from a common two-component system, where a single HK phosphotransfers reversibly to two separate output RRs. We show that under the experimentally observed parameters, this motif implements a sigmoidal signal-response relationship, whereby one of the RRs acts as a phosphate sink towards the other. We identify two mathematical conditions on the system parameters that are necessary for sigmoidality and define key parameters that control threshold levels and sensitivity. We confirm these findings experimentally by *in vitro* reconstitution of the “one HK-two RR” motif found in *S. meliloti*. Particularly, we show that the level of sigmoidality in this system can be experimentally controlled by the amount of sink RR and also through an auxiliary protein, CheS. These findings show that the one HK-two RR motif can open the way to the design of novel threshold systems in synthetic biology.

## Introduction

Cells process external cues in order to produce appropriate responses that ensure survival and efficient proliferation. They achieve this goal through a myriad of signaling and gene regulatory networks, which implement specific signal processing capabilities such as switch-like threshold dynamics, logic gates, oscillations, and noise filtering [1]–[8]. Understanding the architecture and response dynamics of these systems is of fundamental value, providing us with a better insight into cell biology and allowing us to engineer *de novo* biological systems. The field of synthetic biology exploits the understanding and components from natural systems to rationally design synthetic systems that implement specific signaling dynamics. So far, this led to the development of oscillatory systems [9], [10], systems with threshold dynamics [1], [11]–[12] and logic gates [13]–[15]. In most cases, these studies use transcriptional regulation to implement the desired dynamics, while a few studies have explored the possibility of extending synthetic design approaches to signaling networks [16]–[18].

Bacterial systems are particularly attractive for attempting synthetic engineering of signaling networks. Most bacteria and certain eukaryotic microbes and plants utilize the so-called two-component signaling systems for signal transduction [19]–[21]. In their most simple implementation, these systems consist of a histidine protein kinase (HK) and a response regulator (RR). The activity of the HK is controlled in most cases by an environmental stimulus, which controls the rate of autophosphorylation. Once phosphorylated, the HK transfers its phosphoryl group to a cognate RR, which in its phosphorylated form mediates the output of the signaling pathway [21]. The phosphotransfer reaction is at the core of all two-component systems, and regulating its specificity could allow direct control over microbial (and to some extent plant) physiology, as well as creating synthetic signaling systems. Thus, several studies have attempted to decipher the coupling specificity of HK and RR proteins [22]–[26] and have generated chimeric HKs with specified and controllable inputs [27]–[31]. More recently, artificial molecular scaffolds have been used to increase the local concentrations of HK and RR proteins, allowing significant control over the phosphotransfer rate [17].

Generating synthetic systems with specified signal processing capabilities, however, requires a deeper understanding of system properties such as the signal-response relationship they embed. Around the core HK-RR interaction, different two-component systems have diverse architectures, which could underpin specific signal processing capabilities. For example, the commonly observed phosphorelays, where the flow of phosphoryl groups from the HK to the RR is relayed through several proteins, are believed to allow signal integration and specific response properties such as control of noise and ultrasensitivity [32]–[36]. Other architectural features such as presence of a bifunctional or a split HK, formation of specific “dead-end” complexes and also transcriptional feedbacks have been shown to allow ultrasensitivity and even bistability [37]–[39]. Of such different architectural features, one that has not attracted much attention is the “sink” system, where two RRs can compete for the phosphoryl group from a single HK. This architectural motif has been identified in several microbial and plant systems [40]–[44]. In the *Sinorhizobium meliloti* chemotaxis pathway, the two response regulators CheY1 and CheY2 are phosphorylated by their cognate kinase CheA. Of these, only CheY2 in its phosphorylated form can bind to the flagellar motor and control its rotation [40]. Both CheYs can also perform reverse phosphotransfer, where they return the phosphoryl group to CheA. Given its high phosphorylation rate (from HK), low reverse phosphorylation rate (to HK), and the observation that the *S. meliloti* chemotaxis system lacks a dedicated phosphatase, it is proposed that CheY1 acts as a sink that accelerates dephosphorylation of CheY2 [40]. A similar situation is described in the *Rhodobacter sphaeroides* and *Helicobacter pylori* chemotaxis pathways [41], [42] and the yeast osmoregulation pathway [43], [45]. In the latter case, the HK, SLN1 autophosphorylates in response to changes in the membrane structure and phosphorylates two downstream RRs, SSK1 and SKN7. *In vitro* phosphotransfer studies found similar dynamics as in the *S. meliloti* chemotaxis pathway with SKN7 displaying significant reverse phosphotransfer to SLN1, while SSK1 showing no such activity [45]. Interestingly, both SSK1 and SKN7 are functionally active in this system, with SSK1 activating the downstream HOG1 MAP kinase cascade [46], [47] and SKN7 acting as a transcription factor for genes involved in various stress related responses [48], [49].

Here, we use mathematical and experimental approaches to identify the full signal processing capabilities of this two-component system. We first develop a generic model of the one HK – two RR motif and perform both analytical and simulation-based analyses. These reveal that this system is capable of both enhancing signal termination time and implementing a threshold signal-response relationship, i.e. the system displays a sigmoidal signal-response relationship in which the steady state levels of the phosphorylated output RR remains low until a threshold level of signal is crossed. We then verify these dynamics experimentally by *in vitro* re-constitution of the two-component proteins from the chemotaxis pathway of *S. meliloti*. Using this *in vitro* setup, we further show that specific properties of the threshold dynamics can be controlled through the concentrations of the core components, as well as through presence of an auxiliary protein that is known to bind the HK in *S. meliloti* [50]. These findings allow better understanding of the physiological responses mediated by phosphate sink-containing two-component systems in microbes and plants, and will facilitate design of synthetic threshold devices using two-component signalling proteins.

## Results

### Analysis of response dynamics in the one HK – two RR motif

**Figure 1:**
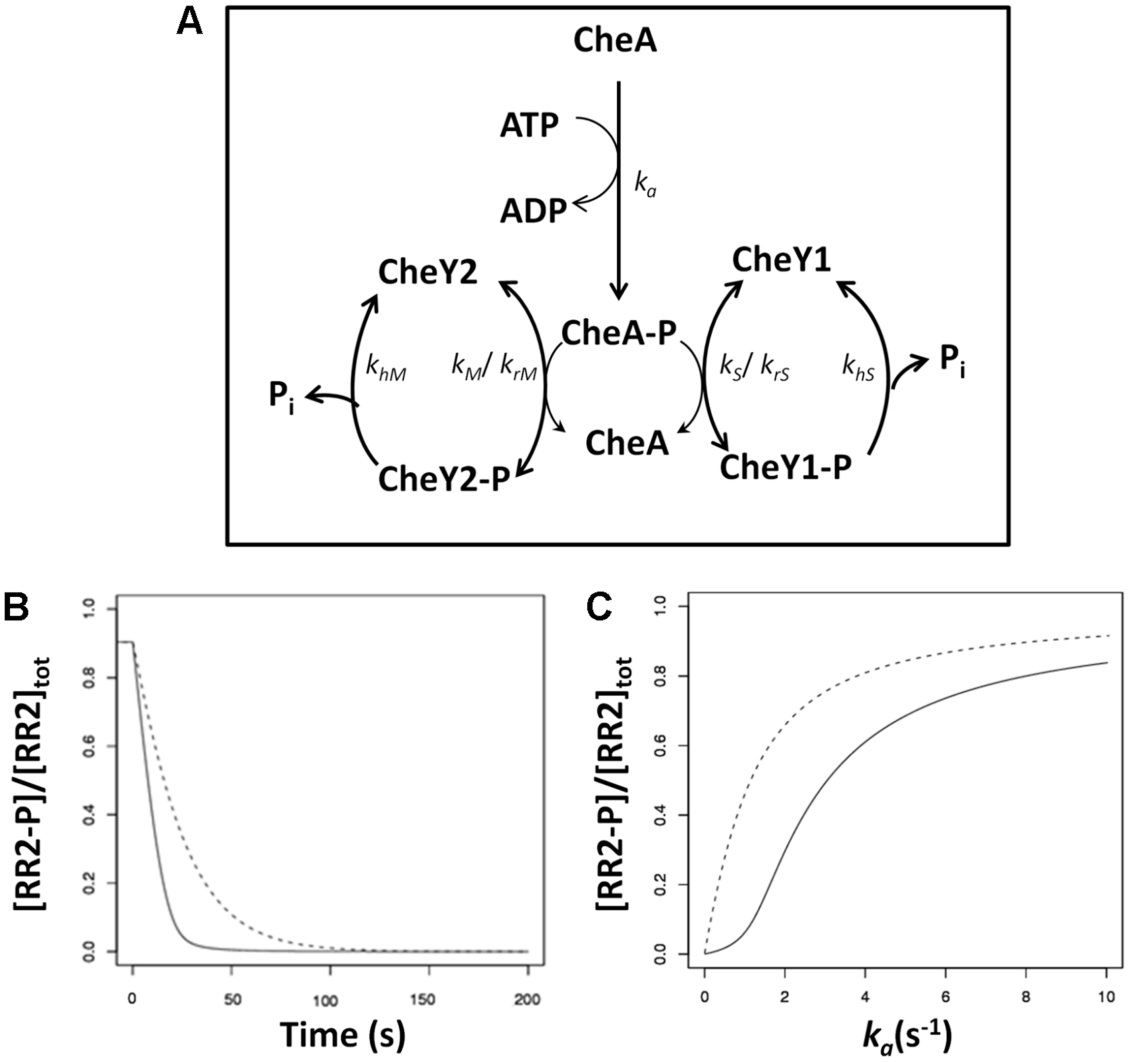
The one HK – two RR motif as seen in the *S. meliloti* chemotaxis signaling pathway (A) A cartoon diagram of the CheA/CheY1/CheY2 system. The diagram is arranged to highlight the role of CheY1 as a phosphate sink for CheY2. Rate constants are shown on the relevant reactions. In the case of reversible reactions, two rate constants are given as *k_forward_* and *k_reverse_*. (**B**) Role of the sink, RR1 (CheY1) in signal termination (i.e. dephosphorylation of RR2 (CheY2)). The x- and y-axis show the time and the corresponding steady state levels of phosphorylated RR2, respectively. A value of *k_a_* was selected that resulted in ∼90% of the total RR2 being phosphorylated at steady state. At t = 0, *k_a_* was reduced to zero and the progress of the reaction to the new steady state simulated. The solid line represents the presence of the sink, while the dashed line shows the absence of the sink. (**C**) Signal-response relationship in the presence (solid line) and absence (dashed line) of sink, RR1 (CheY1). The x- and y-axis show the signal (*k_a_*) and the corresponding steady state level of phosphorylated RR2 (CheY2), respectively.

While the implementation of the phosphate sink motif in diverse two-component systems could differ in the molecular details of the proteins involved and their exact kinetic rates, the sink mechanisms can be formulated as a general architectural motif (Figure 1A and S1A); a two-component system comprising a single HK and two RRs, namely the output-RR and the sink-RR (as referred to, in the rest of the text). We have developed a generic model of this motif and parameterized it using experimental measurements from the reaction kinetics of the *S. meliloti* chemotaxis and yeast osmoregulation systems (see Methods). To monitor temporal dynamics in the presence of a signal, we simulated two conditions, one with the sink-RR and one without the sink-RR. Using the “controlled comparison” approach [51], we simulated each scenario at a signal level that resulted in 90% phosphorylation of the output-RR at steady state. The signal was then removed and the half-time for the decay of phosphorylated output-RR measured. We found that under the experimentally measured parameters, the presence of the sink-RR decreases the half-time for the output-RR dephosphorylation by more than 2-fold in both *S. meliloti* and yeast (Figure 1B and S1B). These simulation results are consistent with previous experimental results [40], which led to the sink hypothesis, and show that in the experimentally observed parameter regime, a sink-RR can accelerate the dephosphorylation of the output-RR.

### The one HK – two RR motif can exhibit a sigmoidal signal-response relationship

Besides temporal dynamics, another key characteristic of any signaling system is the signal-response relationship that it implements, i.e. the steady state output of the system for any given signal level [52]. Focusing again on experimentally measured parameters, we found that the presence of the sink-RR changes the signal-response relationship in the system from hyperbolic to sigmoidal (Figures 1C and S1C). In other words, the presence of the sink-RR allows threshold dynamics in these natural systems, whereby the steady state level of the phosphorylated output-RR remains low until a threshold signal level is reached, at which point the capacity of the sink-RR is filled (Figure S3). Once the sink RR is filled, the steady state level of phosphorylated output-RR is highly sensitive to small changes in signal.

**Figure 2:**
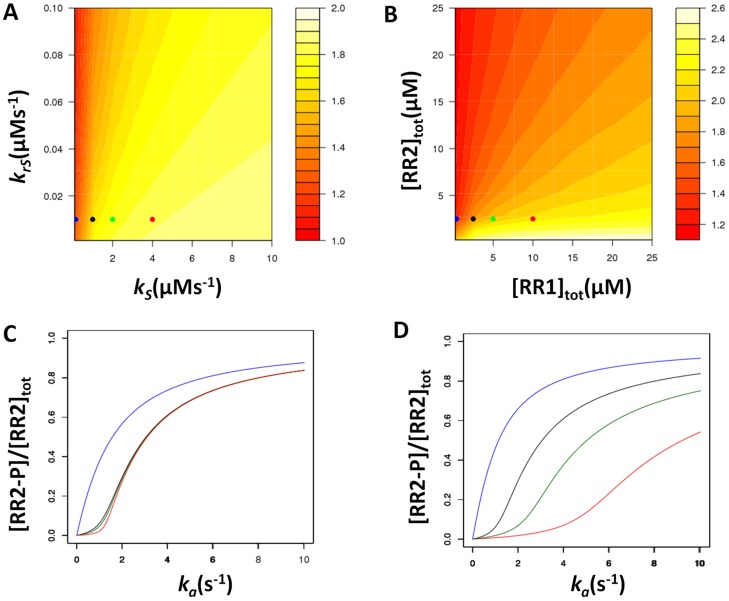
The effect of parameter changes on the “sigmoidality” of the signal-response curve. The level of sigmoidality, Hill coefficient, is shown as a heat map on each panel. (**A**) Effect of varying the forward and reverse phosphotransfer rates for the sink RR (CheY1; x-axis; *k_S_* and y-axis; *k_rS_*). (**B**) Effect of varying the total concentration of the output RR (CheY2; y-axis) and sink RR (CheY1; x-axis). (**C**) Effect of changing the phosphotransfer rate (ks) from CheA to the sink protein (CheY1) on the signal response curve. Each curve is coloured to match the parameter values indicated by the coloured spots on the heatmap shown in panel (A). (**D**) Effect of changing the concentration of the sink protein (CheY1) on the signal response curve. Each curve is coloured to match the parameter values indicated the coloured spots on the heatmap shown in panel (B).

To better understand whether the sensitivity and threshold levels in the sigmoidal signal-response curve can be controlled, and by which parameters, we performed a sensitivity analysis around experimentally measured kinetic rates from *S. meliloti* and yeast (Figures 2, S4, S5 and S2). This revealed several kinetic features for ensuring a sigmoidal signal-response relationship (see below for exact necessary conditions). For example, we found that a key kinetic feature is for phosphotransfer to the sink-RR (parameter *k_S_*) to be faster than reverse phosphotransfer from the sink-RR back to the HK (parameter *k_rS_*). Under this condition, the steady state phosphorylation level of output-RR remains low until the sink-RR is almost fully phosphorylated (Figure S3), resulting in a high level of sigmoidality in the signal-response curve (Figures 2A and S2A). We also found that both the sharpness of the sigmoidal signal-response relationship and the threshold signal level can be controlled through changes in parameters and the ratio of the concentration of HK to the two RRs (Figures S4 and S5). In particular, the phosphotransfer rate constant between the HK and sink-RR (Figures 2A and S2A), and the autodephosphorylation rate constant of the sink-RR (Figures S4 and S2) can affect the sharpness of the signal-response curve, while the threshold signal level is determined by the amount of sink present (Figures 2B and S2B). The effect of the autodephosphorylation rate of the sink-RR can be intuitively understood as increasing this rate directly increases the level of signal required to “fill” the sink-RR. The effect of the forward phosphotransfer rate (between the HK and sink-RR) can be understood when considering the dynamics of the system. When the phosphotransfer from the HK to the two RRs occurs at comparable rates, the increase in the phosphorylation of both RRs occurs in linear fashion. In other words, any increase in the signal levels trickles down the system to affect both RRs. However, what is required from an ultrasensitive signal-response relationship is that one of the RRs remains largely unaffected by increasing signals until a threshold signal is reached. To create such dynamics, having a higher phosphotransfer rate to the other RR is essential, such that any small increases in signal predominantly result in alterations of only this RR.

### Necessary conditions for the one HK – two RR motif to exhibit sigmoidal signal-response relationships

To understand more completely the effects of parameters on the signal-response curve, we derived an analytical description for this curve and computed its second derivative when the signal level is zero (see Text S1). The second derivative at zero can be used as an indicator of sigmoidal or hyperbolic nature of the signal-response relationship; a hyperbolic shape of the signal-response curve implies that the second derivative of this function is constantly negative on its domain (i.e. positive signals), while a sigmoidal shape implies that the second derivative is initially positive and then it changes sign. Thus, the sign of the second derivative of the signal-response curve at zero can be taken as a test for sigmoidality [53]. Using this approach we found two necessary conditions on the parameters of the system for achieving a sigmoidal signal-response relationship (i.e. conditions that are required for a positive second derivative at zero): (*i*) *k_S_·k_hS_·[RR1]_tot_*≠0 *and (ii) k_S_*>*k_rS_*, where *k_hS_* is the autodephosphorylation rate constant of the sink-RR, *[RR1]_tot_* is the total amount of sink-RR, and *k_S_* (*k_M_*) and *k_rS_* (*k_rM_*) are the forward and reverse phosphotransfer rate constants of the sink-RR (output-RR) respectively (see Text S1). The first condition shows that the sink-RR is necessary for the system to exhibit sigmoidality. Provided these two conditions are satisfied and, additionally *k_rS_/k_S_<k_rM_/(k_M_+k_rM_)*, having high concentrations of the HK and the sink-RR (i.e. the RR with no/weak reverse phosphotransfer to the HK), and low concentration of the output-RR further ensures sigmoidality. It is important to note that experimentally measured values from both the *S. meliloti* chemotaxis and yeast osmoregulation systems fit with these analytical conditions for sigmoidality (see Tables 1 and S1). We found that these analytical results on the necessary conditions for the sigmoidality of the signal-response relationship are further simplified when assuming complex formation in the phosphotransfer reactions (Text S1). In particular, the second condition (i.e. of having *k_S_*/*k_rS_*>1) is not a strict requirement for the second derivative of the signal-response curve at zero to attain a positive value. In this extended model, the second necessary condition becomes either *k_S_*/*k_rS_*>1 or *k_S_*/*k_rS_>(k_yM_-k_yS_)/k_yrS_*, where *k_yM_, k_yS_, k_yrS_* are the inverse of the Michaelis-Menten constants of the added complexes in the forward phosphotransfer reactions of the sink-RR and output-RR, and the reverse phosphotransfer reaction of the sink-RR, respectively (see Text S1). We conclude that for sigmoidality to arise, the quotient *k_S_*/*k_rS_* must be larger than a specific value, which depends on the parameters of the system and, further, sigmoidality cannot arise simply by the introduction of complex forming reactions in a system without a sink-RR.

**Table 1:** The parameters used for the model of the *S. meliloti* phosphate sink.

Parameter	Description	Value	Unit	Reference
*k_1_*	Rate constant for formation of the CheA.ATP complex	1	(µMs)^−1^	[40]
*k_2_*	Rate constant for dissociation of the CheA.ATP complex	100	s^−1^	[40]
*k_a_*	Autophosphorylation rate constant (i.e. rate constant for conversion of the CheA.ATP complex into CheA-P+ADP).	Varied	s^−1^	
*k_S_*	CheA-P to CheY1 (sink RR) phosphotransfer	1	(µMs)^−1^	Fitted to data from [40] (see Methods)
*k_rS_*	CheY1-P to CheA Reverse phosphotransfer	0.01	(µMs)^−1^	Fitted to data from [40] (see Methods)
*k_M_*	CheA-P to CheY2 (main RR) phosphotransfer	2	(µMs)^−1^	Fitted to data from [40] (see Methods)
*k_rM_*	CheY2-P to CheA Reverse phosphotransfer	1	(µMs)^−1^	Fitted to data from [40] (see Methods)
*k_hS_*	Autodephosphorylation of CheY1 (sink RR)	0.056	s^−1^	[40]
*k_hM_*	Autodephosphorylation of CheY2 (main RR)	0.066	s^−1^	[40]
[A]tot	Total concentration of CheA	10	µM	see Methods
[Y1]tot	Total concentration of CheY1	2.5	µM	see Methods
[Y2]tot	Total concentration of CheY2	2.5	µM	see Methods

The finding that achieving a sigmoidal signal-response relationship for the single HK- two RR system is facilitated by the presence of complexes, prompted us to use the chemical reaction network toolbox [54] to analytically assess the potential of bistability. We found that when the phosphotransfer reactions are modelled as bi-molecular reactions, the system is not capable of bistability (see Text S2 and S2). However, when considering complex formation and alternative reaction schemes involving the different possible binding events among the HK, the two RRs and their complexes, we found that a certain scenario allows for the presence of bistability in the system (see Text S2 and S3). In this scenario, the HK can bind to both of the RRs, irrespective of its own phosphorylation state and the phosphorylation states of the two RRs. The resulting system contains four complexes between the phosphorylated/unphosphorylated HK and the phosphorylated/unphosphorylated RRs, and can permit bistability under certain parameter regimes (see Text S2 and S3).

### Experimental verification of the sigmoidal signal-response relationship in a one HK – two RR motif

**Figure 3:**
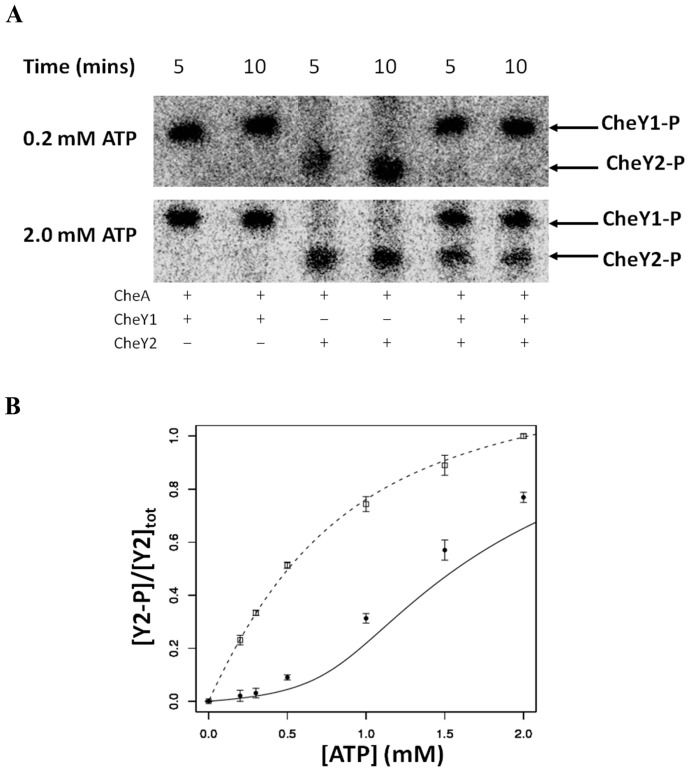
Experimental validation for the role of the sink RR in shaping the signal-response curve. The steady-state level of phosphorylated CheY2 was measured in the presence or absence of the sink (i.e. CheY1) at different ^32^P-ATP concentrations. (**A**) Phosphorimages showing phosphorylated CheY2 levels in the presence or absence of CheY1 at low (0.2 mM) and high (2 mM) ATP levels. The indicated quantity of [γ-^32^P] ATP was added to a reaction mixture containing 10 µM CheA, 2.5 µM CheY2, and where indicated 2.5 µM CheY1. (**B**) Graph comparing the observed steady state levels of phosphorylated CheY2 with and without the sink, CheY1. The phosphorylated CheY2 levels predicted by the model are shown with a dashed line (in absence of sink) and with a solid line (in presence of sink), while the experimentally measured values are shown by squares (in absence of sink) and circles (in presence of sink). Error bars show the standard error of the mean obtained from three independent experiments.

To test the model findings experimentally, we re-constituted *in vitro* the CheA, CheY1 (sink-RR) and CheY2 (output-RR) proteins from *S. meliloti*. *In vivo*, CheA kinase activity is controlled by interaction with the signaling domain of chemoreceptor proteins [55]. Since it is experimentally difficult to re-constitute chemoreceptors in the *in vitro* system, we varied the kinase activity of CheA by varying the concentration of its substrate, ATP, as a proxy for the *in vivo* signal. This allowed us to monitor the steady state levels of phosphorylated CheY1 and CheY2 at different levels of kinase activity, i.e. to derive an experimental signal-response curve. We found excellent quantitative agreement between the signal-response curves resulting from the model and experiments. In the presence (absence) of CheY1, the steady state levels of phosphorylated CheY2 displayed a sigmoidal (hyperbolic) relation with increasing ATP levels (Figure 3). Thus, these experiments strongly suggest that the *S. meliloti* one HK – two RR motif displays a sigmoidal signal-response relationship *in vivo* and could potentially function as a threshold device.

### CheS sharpens the signal-response curve

In the *S. meliloti* system, the behavior of the sink-RR (CheY1) was found to be altered by a small auxiliary protein, CheS [50]. In particular, it was shown that CheY1 binds 100-fold more strongly to the CheA∶CheS complex than to CheA alone and that the decay of phosphorylated CheA (CheA-P) in the presence of CheY1 is faster with CheS than without. This suggests that CheS might directly or indirectly promote CheY1 dephosphorylation and thus make the sink-RR more efficient in allowing signal termination [50]. Analysis of the analytical solution of our model suggests that another way of increasing the efficiency of the sink is to increase the rate at which phosphoryl groups are transferred from CheA to the sink CheY (i.e. by increasing *k_s_*). Moreover, since CheA∶CheS binds CheY1 100-fold more strongly than CheA alone [50], it is conceivable that CheS, in addition to its effects on CheY1-P dephosphorylation, could also accelerate phosphotransfer from CheA-P to CheY1. This would further enhance the possibility of the analytical conditions for sigmoidality to be fulfilled (see above).

**Figure 4:**
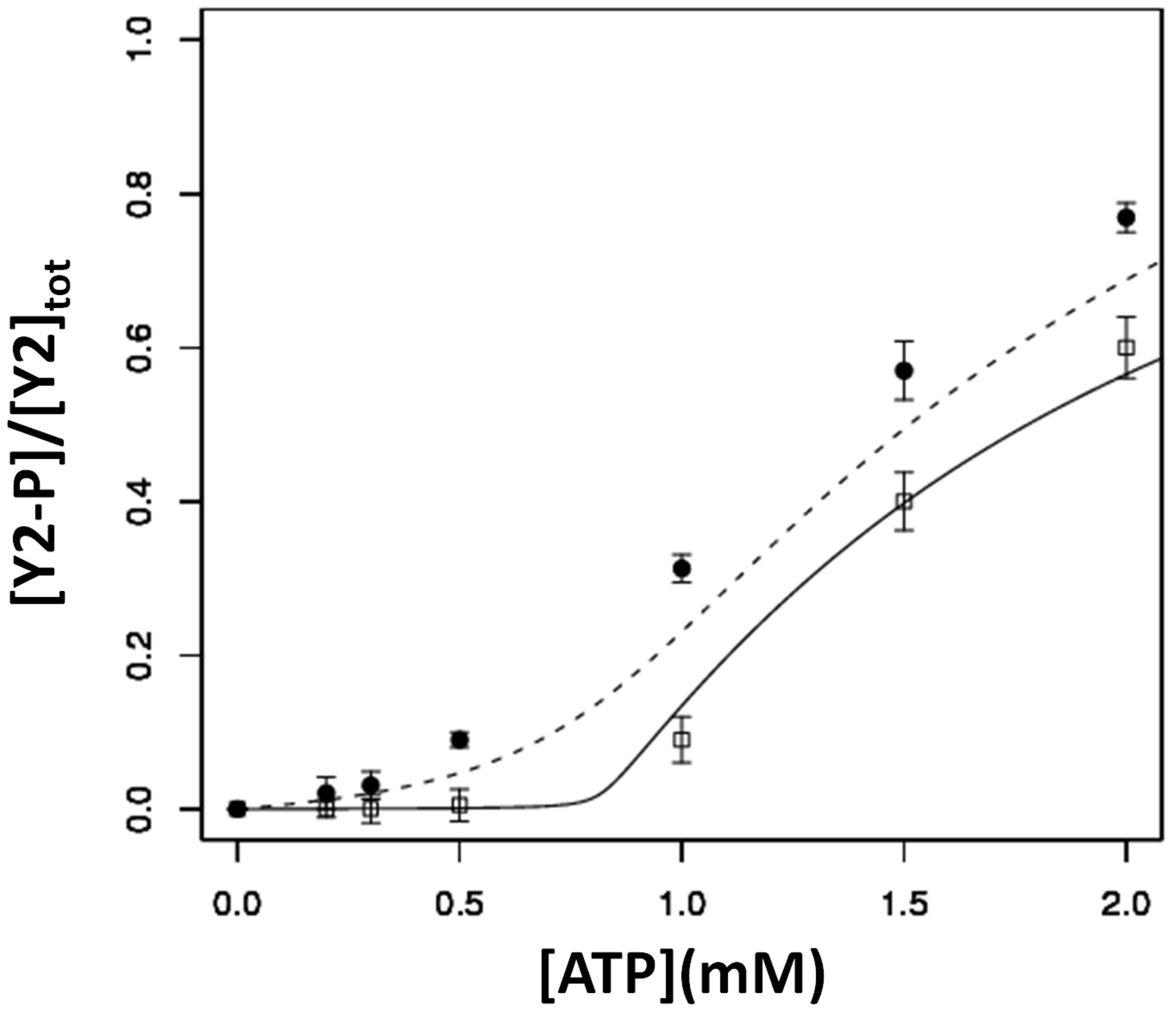
Effect of CheS on the signal-response curve. The x- and y-axis show the ATP level and the corresponding steady state level of phosphorylated CheY2, respectively. The experimentally measured values are shown in circles (absence of CheS) and squares (presence of CheS). The phosphorylated CheY2 levels predicted by the model are shown with a dashed line (absence of CheS) and with a solid line (presence of CheS; where the CheA-P to CheY1 phosphotransfer reaction rate constant (*k_s_*) and CheY1-P dephosphorylation rate constant (*k_hs_*) were optimized for best fit to the experimental data; *k_s_* = 50 and *k_hs_* = 0.067). See Figure S6 for alternative fits to these experimental data where we have individually modelled the effect of CheS altering only *k_s_* or *k_hs_*. Error bars show the standard error of the mean obtained from three independent experiments.

Towards obtaining a better understanding of the role of CheS in the system and quantifying its potential effects on the signal-response curve, we first re-constituted CheS in the *in vitro* assay along with CheA, CheY1 and CheY2. Running phosphotransfer experiments in the presence or absence of CheS, we found that the presence of CheS in the system resulted in the sharpening of the signal-response curve (Figure 4), with the Hill coefficient increasing from 3.43, in the absence of CheS, to 7.61, in the presence of CheS. This increase in the Hill coefficient is in line with the observed capacity of two-component systems to display high levels of ultrasensitivity [36] and potentially bistability [37]–[39]. In an attempt to recapitulate these experimental findings in our mathematical model, we optimised two parameters: the rate of CheY1-P dephosphorylation (*k_hs_*) and/or the rate of phosphotransfer between CheA and CheY1 (*k_s_*). We found that the experimentally observed sharpening of the signal-response curve by CheS can be best recapitulated by increasing both *k_s_* and *k_hs_* (Figure 4), suggesting that CheS may increase both the rate at which CheA-P donates phosphoryl groups to the sink CheY and the rate at which the sink CheY dephosphorylates. These results suggest that the function of CheS is to sharpen the threshold of the sigmoidal signal-response curve given by the system comprising CheA, CheY1 and CheY2.

## Discussion

We have analyzed the system dynamics of an architectural motif found in bacterial two-component signalling pathways where a single HK can reversibly phosphorylate two RRs. We have shown that this one HK-two RR motif can accelerate signal termination, i.e. act as a sink, as hypothesized before [40], but more interestingly, allows the system to exhibit a sigmoidal signal-response relationship. This high level of sigmoidality and corresponding high Hill coefficients can be explained by the effect of the sink-RR on the response dynamics. Due to differing phosphotransfer kinetics, as signal levels increase from zero and kinase activity increases, phosphoryl groups are preferentially given to sink-RR rather than the output-RR (Figure S3). This continues until the sink-RR becomes saturated with phosphoryl groups i.e. is completely phosphorylated. At this threshold point, phosphorylation levels of the output-RR rise dramatically with increasing signal, giving the observed sigmoidal response. We have shown that such threshold behavior is observed under experimentally measured parameters from the *S. meliloti* chemotaxis and yeast osmoregulation pathways. Further, theoretical analyses showed that the presence of a sigmoidal signal-response relationship necessitates two conditions on the system; (*i*) the sink-RR to be present and (*ii*) *k_S_*>*k_rS_*, where *k_S_* and *k_rS_* are the forward and reverse phosphotransfer rate constants of the sink-RR respectively. Factors that promote operation of the sink e.g. increasing the kinetic preference of the kinase for the sink-RR over the output-RR, and/or increasing the rate at which the sink can autodephosphorylate all increase this sigmoidality by sharpening the transition at the threshold point (Figure 4). We verified these findings experimentally, showing that the auxiliary protein, CheS in the *S. meliloti* chemotaxis pathway, can modulate levels of sigmoidality (resulting in Hill coefficients of 7.6) by sharpening the response threshold.

These findings have important implications for understanding bacterial physiology and designing synthetic signaling circuits. In broad terms, the findings of this study will have implications for any two-component signaling circuit where multiple response regulators compete for phosphorylation by a single phosphodonor. This includes the cases where the HK acts as the phosphodonor, as well as the cases where this function is performed by an Hpt domain or protein. These include the majority of bacterial chemotaxis systems (which employ CheY and CheB as response regulators) [40]–[42], fungal osmoregulatory circuits [43] and certain plant signaling systems [44]. Additional examples include the *E. coli* kinases NarX and NarQ that can both phosphorylate the response regulators, NarL and NarP [56]. Similarly, in *Caulobacter crescentus*, the kinases DivJ and PleC can each phosphorylate the two response regulators, DivK and PleD [57]. The present study indicates that these systems might be acting as a threshold device, whereby cells commit to a specific outcome only above certain signal thresholds. Alternatively, the threshold behavior could be used for regulating the noise characteristics of the system [35], [58]. It is important to note however, that the one HK – two RR architectural motif is *able* to display sigmoidal signal-response relationships, but does not preclude hyperbolic relationships. In other words, this motif cannot be taken as proof for threshold behavior but should be taken as indicative and be considered in experimental design when analyzing the response dynamics in associated signaling systems.

Synthetic biology has so far concentrated on designing small circuits based on transcriptional regulation. While two-component systems have been recognized as potential candidates for synthetic design, the main efforts have concentrated on engineering chimeric proteins and interaction specificity [15], [17], [27]–[31]. Our findings show that a system dynamics perspective can allow understanding of the signal processing capabilities of natural bacterial signaling pathways and new avenues for reengineering these. Exploiting the single HK - two RR system in the construction of synthetic signaling circuits will require coupling of an appropriate output (e.g. an RR that can act as a transcription factor) to a useful signal that can control HK activity. This could be accomplished through mutational alterations on the signal and output of an existing natural system (such as the one used here), using chimeric proteins, or by artificially engineering phosphate sinks into existing two-component systems.

Two-component proteins are highly modular, and evolution seems to have exploited this feature in creating diverse architectures in signaling. Studies such as those provided here should allow us to understand these functionalities and ultimately lead to their application in synthetic biology.

## Methods

### A mathematical model for a phosphate sink

To model the one HK - two RR motif, the dynamics was considered in isolation of other cellular components. The reactions in this system that we have included in the model are;
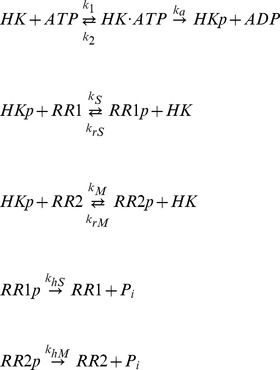
where HK, RR1, and RR2 stand for CheA, CheY1 and CheY2 respectively in the *S. meliloti* chemotaxis system (Figure 1) and for SLN1, SSK1 and SKN7 in the yeast osmoregulation system (Figure S1). The -p suffix represents phosphorylated forms of these proteins. The above reaction scheme can be used to derive a system of ordinary differential equations (ODEs), which describe the changes in concentrations of proteins over time;
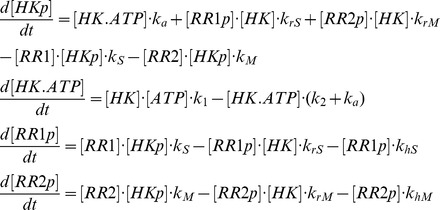



In addition, we have three conservation equations;
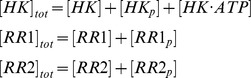



To analyze the behavior of the system with increasing signal, the incoming signals were simulated (e.g. chemoreceptors in case of the chemotaxis system or membrane alterations in the yeast system) as an increase in the autophosphorylation rate constant of the HK (*k_a_*). The model was parameterized with data from literature (see Table 1). In the case of the *S. meliloti* chemotaxis system the parameters for phosphotransfer to CheY1 and CheY2 (*k_S_, k_rS_, k_M_ and k_rM_*) were derived through fitting the simulation data to previously published *in vitro* experiments [40]. Fitting was done using a hybrid genetic algorithm (functions ga and fmincon from the MATLAB Global Optimization Toolbox).

#### Temporal simulations and signal-response curve

The model was numerically integrated to derive time course and steady state signal-response relationships. The latter analysis gives the steady state phosphorylated RR levels at a given signal (*k_a_*), where signal was taken as the rate constant of HK autophosphorylation and allows deriving a so-called signal-response curve. This curve is found by numerically integrating the system to steady state at a fixed signal level and then numerically “following” this steady state, while changing the signal. This analysis is equivalent to allowing the system to reach steady state under different signal values. Both time course and signal-response analyses were performed using the software packages XPPAUT (http://www.math.pitt.edu/~bard/xpp/xpp.html) and Oscill8 (http://oscill8.sourceforge.net). An explicit description of the inverse of the signal-response curve was also obtained, using a recently developed recursive technique [35], [59] (see Text S1). The resulting analytical function for the signal-response curve was then used to verify the results of the numerical approach and to derive the necessary conditions that the parameters must fulfill for the signal-response curve to be sigmoidal. This analytical approach is also used to extend the analysis to the case with complex formation (see Text S1).

#### Measuring “sigmoidality” of signal-response curves and sensitivity of this feature to parameters

To measure sigmoidality of the signal-response curve the Hill coefficient was used as previously described [60], [61]. The Hill coefficient is measured as ln81/ln(S90/S10) where S90 and S10 are the signal levels for achieving 90 and 10 percent of output saturation, respectively. Using alternative measures, such as the maximum value of the response coefficient across the signal domain produces qualitatively similar results as those shown in Figures 2, S4 and S5. To quantify the sensitivity of sigmoidality of the signal-response curve to variations in each of the parameters, these were varied from their described experimentally measured values (Table 1) and in a biologically relevant range. Each parameter was varied around its basic value up/down 10-fold and the Hill coefficient of the resulting signal-response curves measured as described above.

#### Experimental design

The CheA, CheY1, and CheY2 system was reconstituted *in vitro* to measure the signal-response curve in the presence/absence of CheY1. For this, phosphorylated CheY2 levels were measured under increasing ATP levels as a proxy for signal. The protein concentrations used for these experiments were 10 µM, 2.5 µM, 2.5 µM for CheA, CheY1 and CheY2 respectively. This gives a ratio of 4∶1∶1, which is different from the *in vivo* measured ratio of 1.5∶20∶20 [40], but the higher HK concentration gave increased capacity to measure CheY1 and CheY2 phosphorylation levels at low levels of signal. We found that the system can display sigmoidality under a range of ratios of sink- and output-RRs to HK, including the experimentally measured ratio, but the level of sigmoidality is altered by this ratio (Figure S5).

#### Plasmids and strains

See Table 2 for the plasmids and strains used. *E. coli* strains were grown in LB medium at 37°C. Antibiotics were used at concentrations of 100 µg ml^−1^ for ampicillin, 34 µg ml^−1^ for chloramphenicol and 25 µg ml^−1^ for kanamycin, where needed. *E. coli* M15pREP4 cells were made competent using the calcium chloride technique [62]. Transformations were performed according to [63].

**Table 2:** The strains and plasmids used in this study.

Strains/plasmid	Description	Source/Reference
*E. coli* strain M15pREP4	Expression host containing pREP4; kanamycin resistant	Qiagen
pQE30	IPTG inducible expression vector. Introduces RGS(H)_6_ at the N terminus of the expressed protein. Confers ampicillin resistance	Qiagen
pQE60	IPTG inducible expression vector. Introduces RGS(H)_6_ at the C terminus of the expressed protein. Confers ampicillin resistance	Qiagen
pRU1735 (pQE60Y1)	Plasmid for overexpressing C-terminally His-tagged CheY1 from *S. meliloti*. pQE60 derivative	[41]
pRU1736 (pQE60Y2)	Plasmid for overexpressing C-terminally His-tagged CheY2 from *S. meliloti*. pQE60 derivative	[41]
pRU1742	Plasmid for overexpressing N-terminally His-tagged CheA from *S. meliloti*. pQE30 derivative	[41]
pBS174 (pET27bmodA/S)	Plasmid for coexpressing *S. meliloti* N-terminally His-tagged CheA and CheS. pET27bmod derivative	[50]

#### Protein purification

His-tagged *S. meliloti* CheA, CheA∶CheS, CheY1 and CheY2 proteins were purified as described previously [50]. Protein purity and concentration was measured as described in [64]. Purified proteins were stored at −20°C.

#### Preparation of CheA-^32^P and CheA-^32^P∶CheS

CheA-^32^P and CheA-^32^P∶CheS were phosphorylated using [γ-^32^P] ATP and purified as described before [65], but with the following modifications: Proteins were phosphorylated in reactions performed at 20°C in phosphotransfer buffer (50 mM Tris HCl, 10% (v/v) glycerol, 5 mM MgCl_2_, 150 mM NaCl, 50 mM KCl, 1 mM DTT, pH 8.0). The final reaction volumes were 2 ml. Reactions were initiated by addition of 2 mM [γ-^32^P] ATP (specific activity 14.8 GBq mmol^−1^; PerkinElmer). After 1 hour incubation, samples were purified by using Ni-NTA columns (Qiagen) as described previously [66]. This purification step removed the unincorporated ATP from the CheA-^32^P and CheA-^32^P∶CheS preparation. Purified proteins were stored at −20°C.

#### Measurement of CheY2-P at different ^32^P- ATP concentrations with and without CheS

Assays were performed at 20°C in phosphotransfer buffer. Either CheA (10 µM) or CheA∶CheS (10 µM) was added to a mixture of 2.5 µM CheY1 and 2.5 µM CheY2 under different ATP concentrations. Following the addition of ^32^P -ATP, reaction aliquots of 10 µl were taken at the indicated time points and quenched immediately in 10 µl of 2× SDS-PAGE loading dye (7.5% (w/v) SDS, 90 mM EDTA, 37.5 mM Tris HCl, 37.5% glycerol, 3% (v/v) β-mercaptoethanol, pH 6.8). Quenched samples were analyzed using SDS-PAGE and phosphorimaging as described previously [67].

## Supporting Information

Figure S1 SSK1 is a phosphate sink for SLN7 in the yeast osmoregulation pathway (A) A cartoon diagram of the SLN1-YPD1-SSK1-SKN7 system. The diagram is arranged to highlight the role of the SSK1 as a phosphate sink for SKN7. Rate constants are shown on the relevant reactions. In the case of reversible reactions, two rate constants are given as *k_forward_* and *k_reverse_*. (B) Role of the sink RR (SSK1) in dephosphorylation of SKN7-P (RR2-P). The x- and y-axis show the time and the corresponding phosphorylated RR2 (SKN7-P) level at steady-state respectively. A value of *k_a_* was selected that resulted in ∼90% of the total RR2 being phosphorylated at steady state. At t = 0, *ka* was reduced to zero and the progress of the reaction to the new steady state was simulated. Solid line represents the presence of the sink (i.e. SSK1), while dashed line shows the absence of the sink. (C) Signal- response curve in the presence (solid line) and absence (dashed line) of the sink RR (SSK1). The x- and y-axis show the signal (*k_a_*) level and the corresponding steady state level of phosphorylated SKN7 (RR2-P) respectively.
(TIF)

Figure S2 Effect of varying the key parameters in the yeast osmoregulation system on the shape of the signal-response curve. The x- and y-axis show the signal (*k_a_*) level and the corresponding level of phosphorylated output RR (SKN7-P) at steady state respectively. Each panel shows a signal-response curve for different parameter values. The results of the basic model are shown in black. The arrow on each panel indicates increasing values of the changed parameter. (**A**) The forward phosphotransfer rate (*k_S_*) for the sink RR was varied from basic model value (of 66.67 µMs^−1^) to 660, and 0. (**B**) Concentration of the sink RR was set to 0 µM, 1.5 µM (basic model) and 3 µM. (**C**) The rate of auto- dephosphorylation of sink RR-P (*k_hS_*) was set to 0 s^−1^, 0.5 s^−1^ (basic model) and 1 s^−1^. (**D**) The forward phosphotransfer rate (*k_M_*) for the main RR, was set to 1 µMs^−1^ (basic model), 0.5 µMs^−1^, and 10 µMs^−1^.
(TIF)

Figure S3 Signal-response relationship for the sink RR and the output RR in the *S. meliloti* system. The x- and y-axis show the signal (*k_a_*) level and the corresponding steady state level of either phosphorylated sink (blue line) or main RR (black line).
(TIF)

Figure S4 The effect of parameter changes on the signal-response curve of the *S. meliloti* system. The signal-response curve Hill coefficient is shown on each panel as a heat map. (**A**) Effect of varying the auto-dephosphorylation rate of the output RR (*k_hM_*; y-axis) and sink RR (*k_hS_*; x-axis). (**B**) Effect of varying the forward phosphotransfer rates to the output and sink RR (*k_M_* and *k_S_*). (**C**) Effect of varying the forward and reverse phosphotransfer rates to the output RR (CheY2; x-axis; *k_M_* and y-axis; *k_rM_*). (**D–F**) Signal-response curves for models corresponding to parameter values indicated as colored circles on the heat maps above; the black circle represents the basic model.
(TIF)

Figure S5 Effect of the stoichiometric ratio of CheA to CheY1, and CheY2 total concentrations on the shape of the signal-response curve for the *S. meliloti* system. (**A**) The signal-response curve Hill coefficient is shown as a heat map. The x-axis shows the total concentration of CheA, while the y-axis shows the total concentration of CheY1 and CheY2 (where [CheY1]tot = [CheY2]tot). (**B**) The signal-response curves resulting from the stoichiometric ratios considered in the *in vitro* experimental system (10∶2.5∶2.5), in black, and the measured values from *S. meliloti* (1.5∶20∶20), in red. The corresponding Hill coefficients are 1.75 and 1.59, respectively.
(TIF)

Figure S6 Effect of CheS on the signal-response curve. On each panel, the x- and y-axis show the ATP level and the corresponding steady state phosphorylated CheY2 levels, respectively. The phosphorylated CheY2 levels predicted by the model are shown with a dashed line (absence of CheS) and with a solid line (presence of CheS), while the experimentally measured values are shown in circles and squares on respective graph. Error bars show the standard error of the mean obtained from three independent experiments. Panel **A** shows the model prediction when only the forward phosphotransfer rate to CheY1 rate is optimized (*k_s_*), while panel **B** shows model prediction when only the CheY1 autodephosphorylation rate (*k_hs_*) is optimized.
(TIF)

Table S1 The parameters used for the model of the yeast phosphate sink.
(DOC)

Text S1 The main supplementary text. Sections 1 and 2 of this file contain the mathematical analysis of a two-component system with one histidine kinase HK and two response regulators RR. Section 3 describes the mathematical model for the two-component system regulating yeast osmoregulation.
(PDF)

Text S2 This file contains the results of the analysis using the Chemical Reaction Network toolbox for the reaction system described in the basic model without complex formation.
(DOC)

Text S3 This file contains the results of the analysis using the Chemical Reaction Network toolbox for the reaction system with formation of complexes during phosphotransfer reactions.
(DOC)

Text S4 This file contains the ordinary differential equation model for the *S. meliloti* system as described in the main text. The file (.ode extension) is compatible with the XPPAUT and other modeling software.
(ODE)

## References

[1] Buchler NE, Cross FR (2009) Protein sequestration generates a flexible ultrasensitive response in a genetic network. Mol Syst Bio 5: 272. 19455136 10.1038/msb.2009.30 PMC2694680

[2] Tyson JJ, Novak B eds. (2012) Irreversible transitions, bistability and checkpoint controls in the eukaryotic cell cycle: a systems-level understanding. *Elsevier*, San Diego, CA.

[3] Tyson JJ, Novak B (2010) Functional motifs in biochemical reaction networks. Annu Rev Phys Chem 61: 219–240. 20055671 10.1146/annurev.physchem.012809.103457 PMC3773234

[4] Battogtokh D, Tyson JJ (2004) Bifurcation analysis of a model of the budding yeast cell cycle. Chaos 14: 653–661. 15446975 10.1063/1.1780011

[5] Novak B, Tyson JJ (2008) Design principles of biochemical oscillators. Nat Rev Mol Cell Biol 9: 981–991. 18971947 10.1038/nrm2530 PMC2796343

[6] Tyson JJ, Albert R, Goldbeter A, Ruoff P, Sible JC (2008) Biological switches and clocks. J R Soc Interface 5: S1–S8. 18522926 10.1098/rsif.2008.0179.focus PMC2706456

[7] Kaimachnikov NP, Kholodenko BN (2009) Toggle switches, pulses and oscillations are intrinsic properties of the Src activation/deactivation cycle. FEBS J 276: 4102–18. 19627364 10.1111/j.1742-4658.2009.07117.x PMC2924194

[8] Chickarmane V, Kholodenko BN, Sauro HM (2007) Oscillatory dynamics arising from competitive inhibition and multisite phosphorylation. J Theor Biol 244 (1) 68–76. 16949102 10.1016/j.jtbi.2006.05.013

[9] Elowitz MB, Leibler S (2000) A synthetic oscillatory network of transcriptional regulators. Nature 403: 335–338. 10659856 10.1038/35002125

[10] Koseska A, Volkov E, Kurths J (2011) Synthetic multicellular oscillatory systems: controlling protein dynamics with genetic circuits. Physica Scripta 84 (4) 045007.

[11] Hsu C, et al (2012) Stochastic signalling rewires the interaction map of a multiple feedback network during yeast evolution. Nat Commun 3: 682. 22353713 10.1038/ncomms1687 PMC3293423

[12] Khalil AS, Lu TK, Bashor CJ, Ramirez CL, Pyenson NC, Joung JK, Collins JJ (2012) A synthetic biology framework for programming eukaryotic transcription functions. Cell 150: 647–658. 22863014 10.1016/j.cell.2012.05.045 PMC3653585

[13] Gardner TS, Cantor CR, Collins JJ (2000) Construction of a genetic toggle switch in *Escherichia coli* . Nature 403: 339–342. 10659857 10.1038/35002131

[14] Siuti P, Yazbek J, Lu TK (2013) Synthetic circuits integrating logic and memory in living cells. Nat Biotech 31: 448–452. 10.1038/nbt.2510 23396014

[15] Friedland AE, et al (2009) Synthetic gene networks that count. Science 324: 1199–1202. 19478183 10.1126/science.1172005 PMC2690711

[16] Wang B, Kitney RI, Joly N, Buck M (2011) Engineering modular and orthogonal genetic logic gates for robust digital-like synthetic biology. Nat Commun 2: 508. 22009040 10.1038/ncomms1516 PMC3207208

[17] Whitaker WR, Davis SA, Arkin AP, Dueber JE (2012) Engineering robust control of two-component system phosphotransfer using modular scaffolds. Proc Natl Acad Sci USA 109: 18090–18095. 23071327 10.1073/pnas.1209230109 PMC3497815

[18] Peisajovich SG, Garbarino JE, Wei P, Lim WA (2010) Rapid diversification of cell signaling phenotypes by modular domain recombination. Sci Signal 328 (5976) 368. 10.1126/science.1182376 PMC2975375 20395511

[19] Wuichet K, Zhulin IB (2010) Origins and diversification of a complex signal transduction system in prokaryotes. Sci Signal 3: ra50. 20587806 10.1126/scisignal.2000724 PMC3401578

[20] Hamer R, Chen PY, Armitage JP, Reinert G, Deane CM (2010) Deciphering chemotaxis pathways using cross species comparisons. BMC Sys Biol 4: 3. 10.1186/1752-0509-4-3 PMC2829493 20064255

[21] Stock AM, Robinson VL, Goudreau PN (2000) Two-component signal transduction. Annu Rev Biochem 69: 183–215. 10966457 10.1146/annurev.biochem.69.1.183

[22] Casino P, Vicente R, Alberto M (2009) Structural insight into partner specificity and phosphoryl transfer in two-component signal transduction. Cell 139: 325–336. 19800110 10.1016/j.cell.2009.08.032

[23] Scott KA, et al (2010) Specificity of localization and phosphotransfer in the CheA proteins of *Rhodobacter sphaeroides* . Mol Microbiol 76: 318–330. 20525091 10.1111/j.1365-2958.2010.07095.x

[24] Podgornaia AI, Laub MT (2013) Determinants of specificity in two-component signal transduction. Curr Opin Microbiol 16: 156–62. 23352354 10.1016/j.mib.2013.01.004

[25] Skerker JM, et al (2008) Rewiring the specificity of two-component signal transduction systems. Cell 133: 1043–54. 18555780 10.1016/j.cell.2008.04.040 PMC2453690

[26] Weigt M, White RA, Szurmant H, Hoch JA, Hwa T (2009) Identification of direct residue contacts in protein-protein interaction by message passing. PNAS 106 (1) 67–72. 19116270 10.1073/pnas.0805923106 PMC2629192

[27] Ninfa AJ (2010) Use of two-component signal transduction systems in the construction of synthetic genetic networks. Curr Opin Microbiol 13 (2) 240–245. 20149718 10.1016/j.mib.2010.01.003 PMC3547608

[28] Levskaya A, et al (2005) Synthetic biology: engineering *Escherichia coli* to see light. Nature 438: 441–442. 16306980 10.1038/nature04405

[29] Moon TS, et al (2011) Construction of a genetic multiplexer to toggle between chemosensory pathways in *Escherichia coli* . J Mol Biol 406 (2) 245–227. 10.1016/j.jmb.2010.12.019 PMC3033806 21185306

[30] Yoshida T, Phadtare S, Inouye M (2007) The design and development of Tar-EnvZ chimeric receptors. Methods Enzymol 423: 166–83. 17609131 10.1016/S0076-6879(07)23007-1

[31] Möglich A, Ayers RA, Moffat K (2009) Design and signaling mechanism of light-regulated histidine kinases. J Mol Biol 385 (5) 1433–44. 19109976 10.1016/j.jmb.2008.12.017 PMC3527124

[32] Bischofs IB, Hug JA, Liu AW, Wolf DM, Arkin AP (2009) Complexity in bacterial cell-cell communication: quorum signal integration and subpopulation signaling in the *Bacillus subtilis* phosphorelay. Proc Natl Acad Sci USA 106: 6459–6464. 19380751 10.1073/pnas.0810878106 PMC2672556

[33] Csikász-Nagy A, Cardelli L, Soyer OS (2010) Response dynamics of phosphorelays suggest their potential utility in cell signalling. J R Soc Interface 8 (57) 480–488. 20702449 10.1098/rsif.2010.0336 PMC3061117

[34] Jatin N, Devi SN, Fujita M, Igoshin OA (2012) Ultrasensitivity of the *Bacillus subtilis* sporulation decision. Proc Natl Acad Sci USA 109: 20196–20197. 10.1073/pnas.1213974109 PMC3528541 23169620

[35] Knudsen M, Feliu E, Wiuf C (2012) Exact analysis of intrinsic qualitative features of phosphorelays using mathematical models. J Theor Biol 300: 01.007. 10.1016/j.jtbi.2012.01.007 22266661

[36] Arnaud C, et al (2010) Broadly heterogeneous activation of the master regulator for sporulation in *Bacillus subtilis* . Proc Natl Acad Sci USA 107: 8486–8491. 20404177 10.1073/pnas.1002499107 PMC2889527

[37] Amin M, Porter SL, Soyer OS (2013) Split histidine kinases enable ultrasensitivity and bistability in two-component signaling networks. PLoS Comp Biol 9 (3) e1002949. 10.1371/journal.pcbi.1002949 PMC3591291 23505358

[38] Igoshin OA, Alves R, Savageau MA (2008) Hysteretic and graded responses in bacterial two-component signal transduction. Mol Microbiol 68: 1196–1215. 18363790 10.1111/j.1365-2958.2008.06221.x PMC4372536

[39] Tiwari A, Ray JC, Narula J, Igoshin OA (2011) Bistable responses in bacterial genetic networks: designs and dynamical consequences. Math Biosci 231: 76–89. 21385588 10.1016/j.mbs.2011.03.004 PMC3095517

[40] Sourjik V, Schmitt R (1998) Phosphotransfer between CheA, CheY1, and CheY2 in the chemotaxis signal transduction chain of *Rhizobium meliloti* . Biochem 37: 2327–2335. 9485379 10.1021/bi972330a

[41] Tindall MJ, Porter SL, Maini PK, Armitage JP (2010) Modeling chemotaxis reveals the role of reversed phosphotransfer and a bi-functional kinase-phosphatase. PLoS Comp Biol 6: e1000896. 10.1371/journal.pcbi.1000896 PMC2924250 20808885

[42] Jiménez-Pearson MA, Delany I, Scarlato V, Beier D (2005) Phosphate flow in the chemotactic response system of *Helicobacter pylori* . Microbiol 151 (10) 3299–3311. 10.1099/mic.0.28217-0 16207913

[43] Posas F, et al (1996) Yeast HOG1 MAP kinase cascade is regulated by a multistep phosphorelay mechanism in the SLN1-YPD1-SSK1 “two-component” osmosensor. Cell 86: 865–875. 8808622 10.1016/s0092-8674(00)80162-2

[44] Lohrmann J, Harter K (2002) Plant two-component signaling systems and the role of response regulators. Plant Physiol 128: 363–369. 11842140 10.1104/pp.010907 PMC1540209

[45] Fabiola J-S, Cook PF, West AH (2005) Kinetic analysis of YPD1-dependent phosphotransfer reactions in the yeast osmoregulatory phosphorelay system. Biochem 44 (1) 377–86. 15628880 10.1021/bi048433s

[46] Posas F, Saito H (1998) Activation of the yeast SSK2 MAP kinase kinase kinase by the SSK1 two-component response regulator. EMBO J 17: 1385–1394. 9482735 10.1093/emboj/17.5.1385 PMC1170486

[47] Horie T, Tatebayashi K, Yamada R, Saito H (2008) Phosphorylated Ssk1 prevents unphosphorylated Ssk1 from activating the Ssk2 MAP kinase kinase kinase in the yeast HOG osmoregulatory pathway. Mol Cell Biol 28: 5172–5183. 18573873 10.1128/MCB.00589-08 PMC2519728

[48] Brown JL, Bussey H, Stewart RC (1994) Yeast Skn7p functions in a eukaryotic two-component regulatory pathway. EMBO J 13: 5186–5194. 7957083 10.1002/j.1460-2075.1994.tb06849.x PMC395467

[49] Krems B, Charizanis C, Entian KD (1996) The response regulator-like protein Pos9/Skn7 of *Saccharomyces cerevisiae* is involved in oxidative stress resistance. Curr Genet 29: 327–334. 8598053 10.1007/BF02208613

[50] Dogra G, et al (2012) *Sinorhizobium meliloti* CheA complexed with CheS exhibits enhanced binding to CheY1 resulting in accelerated CheY1-P dephosphorylation. J Bacteriol 194 (5) 1075–1087. 22194454 10.1128/JB.06505-11 PMC3294773

[51] Alves R, Savageau MA (2000) Extending the method of mathematically controlled comparison to include numerical comparisons. Bioinformatics 16: 786–798. 11108701 10.1093/bioinformatics/16.9.786

[52] Tyson JJ, Katherine CC, Novak B (2003) Sniffers, buzzers, toggles and blinkers: dynamics of regulatory and signaling pathways in the cell. Curr Opin Cell Biol 15: 221–231. 12648679 10.1016/s0955-0674(03)00017-6

[53] Kothamachu VB, Feliu E, Wiuf C, Cardelli L, Soyer OS (2013) Phosphorelays provide tunable signal processing capabilities for the cell. PLoS Comp Biol 9 (11) e1003322. 10.1371/journal.pcbi.1003322 PMC3820541 24244132

[54] Hazelbauer GL, Falke JJ, Parkinson JS (2008) Bacterial chemoreceptors: high-performance signaling in networked arrays. Trends Biochem Sci 33 (1) 9–19. 18165013 10.1016/j.tibs.2007.09.014 PMC2890293

[55] Shinar G, Feinberg M (2012) Concordant chemical reaction networks and the species-reaction graph. Math Biosci 241 (1) 1–23. 22940368 10.1016/j.mbs.2012.08.002 PMC4701587

[56] Noriega CE, Hsia-Yin L, Li-Ling C, Williams SB, Stewart V (2010) Asymmetric cross-regulation between the nitrate-responsive NarX-NarL and NarQ-NarP two-component regulatory systems from *Escherichia coli* K-12. Mol Microbiol 75 (2) 394–412. 19968795 10.1111/j.1365-2958.2009.06987.x PMC3034140

[57] Paul R, et al (2008) Allosteric regulation of histidine kinases by their cognate response regulator determines cell fate. Cell 133 (3) 452–61. 18455986 10.1016/j.cell.2008.02.045 PMC2804905

[58] Shibata T, Koichi F (2005) Noisy signal amplification in ultrasensitive signal transduction. Proc Natl Acad Sci USA 102 (2) 331–336. 15625116 10.1073/pnas.0403350102 PMC544281

[59] Feliu E, Knudsen M, Andersen LN, Wiuf C (2012) An algebraic approach to signaling cascades with N layers. Bull Math Biol 74: 45–72. 21523510 10.1007/s11538-011-9658-0

[60] Zhang Q, Bhattacharya S, Andersen ME (2013) Ultrasensitive response motifs: basic amplifiers in molecular signalling networks. Open Biol rsob.130031. 10.1098/rsob.130031 PMC3718334 23615029

[61] Goldbeter A, Koshland DE (1981) An amplified sensitivity arising from covalent modification in biological systems. Proc Natl Acad Sci USA 78: 6840–6844. 6947258 10.1073/pnas.78.11.6840 PMC349147

[62] Sambrook J Russell JB (2001) Molecular cloning: a laboratory manual. Cold Spring Harbor, NY, USA: Cold Spring Harbor Laboratory Press.

[63] Hanahan D (1983) Studies on transformation of Escherichia coli with plasmids. J Mol Biol 166: 557. 6345791 10.1016/s0022-2836(83)80284-8

[64] Porter SL, Armitage JP (2002) Phosphotransfer in *Rhodobacter sphaeroides* chemotaxis. J Mol Biol 324 (1) 35–45. 12421557 10.1016/s0022-2836(02)01031-8

[65] Porter SL, Roberts MAJ, Manning CS, Armitage JP (2008) A bifunctional kinase-phosphatase in bacterial chemotaxis. Proc Natl Acad Sci USA 105: 18531–18536. 19020080 10.1073/pnas.0808010105 PMC2587623

[66] Porter SL, Wadhams GH, Armitage JP (2007) *In vivo* and *in vitro* analysis of the *Rhodobacter sphaeroides* chemotaxis signaling complexes. Methods Enzymol 423: 392–413. 17609142 10.1016/S0076-6879(07)23018-6

[67] Porter SL, Warren AV, Martin AC, Armitage JP (2002) The third chemotaxis locus of Rhodobacter sphaeroides is essential for chemotaxis. Mol Microbiol 46: 1081–1094. 12421313 10.1046/j.1365-2958.2002.03218.x

