# Supplementary figures and images for: Phosphate Sink Containing Two-Component Signaling Systems as Tunable Threshold Devices
Source: PLoS Comput Biol. 2014 Oct 30;10(10):e1003890. doi: 10.1371/journal.pcbi.1003890 (PMC4214558; doi:10.1371/journal.pcbi.1003890)

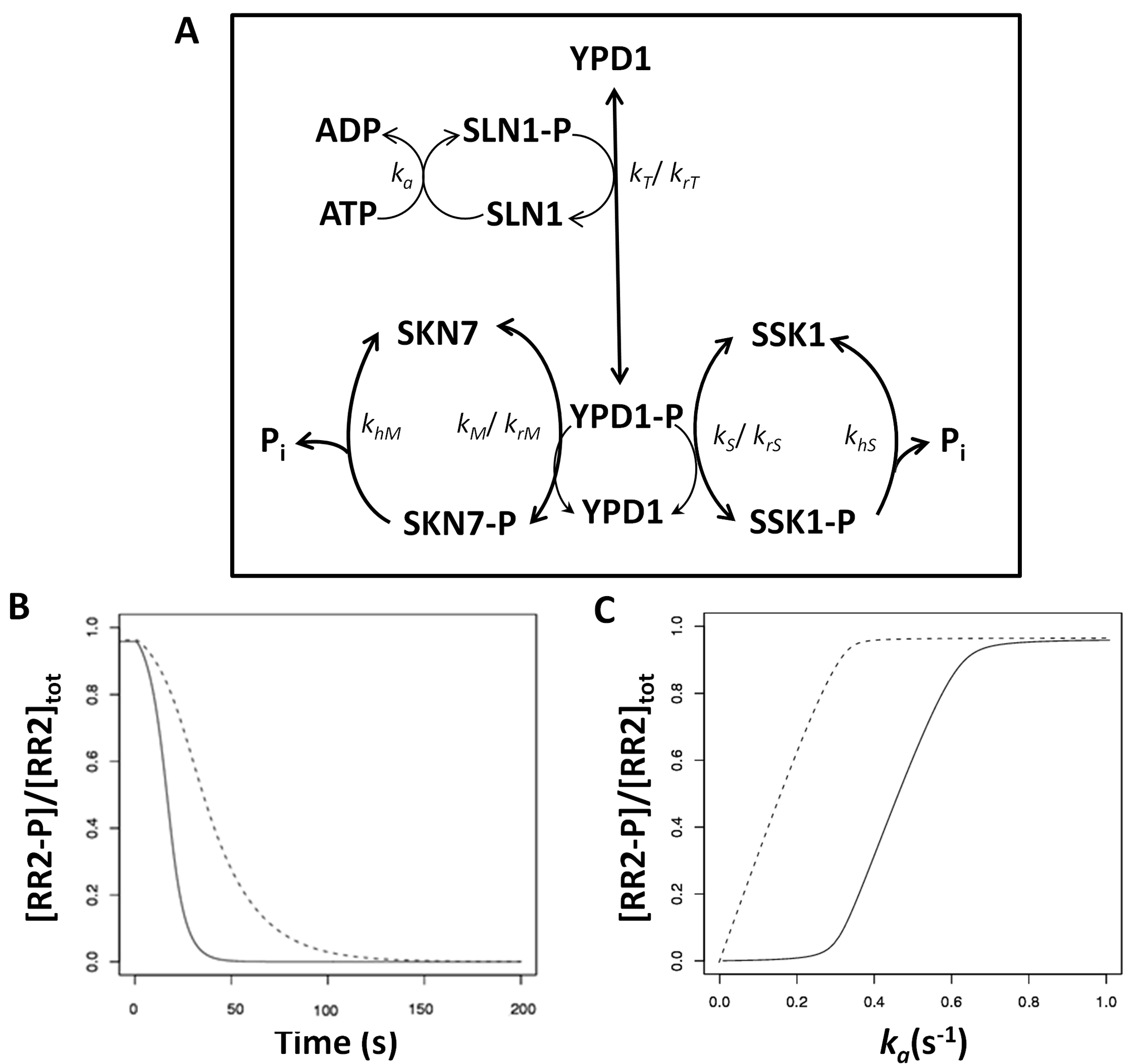

Supplement: Figure S1 — SSK1 is a phosphate sink for SLN7 in the yeast osmoregulation pathway (A) A cartoon diagram of the SLN1-YPD1-SSK1-SKN7 system. The diagram is arranged to highlight the role of the SSK1 as a phosphate sink for SKN7. Rate constants are shown on the relevant reactions. In the case of reversible reactions, two rate constants are given as kforward and kreverse. (B) Role of the sink RR (SSK1) in dephosphorylation of SKN7-P (RR2-P). The x- and y-axis show the time and the corresponding phosphorylated RR2 (SKN7-P) level at steady-state respectively. A value of ka was selected that resulted in ∼90% of the total RR2 being phosphorylated at steady state. At t = 0, ka was reduced to zero and the progress of the reaction to the new steady state was simulated. Solid line represents the presence of the sink (i.e. SSK1), while dashed line shows the absence of the sink. (C) Signal- response curve in the presence (solid line) and absence (dashed line) of the sink RR (SSK1). The x- and y-axis show the signal (ka) level and the corresponding steady state level of phosphorylated SKN7 (RR2-P) respectively. (TIF) [file pcbi.1003890.s001.tif]

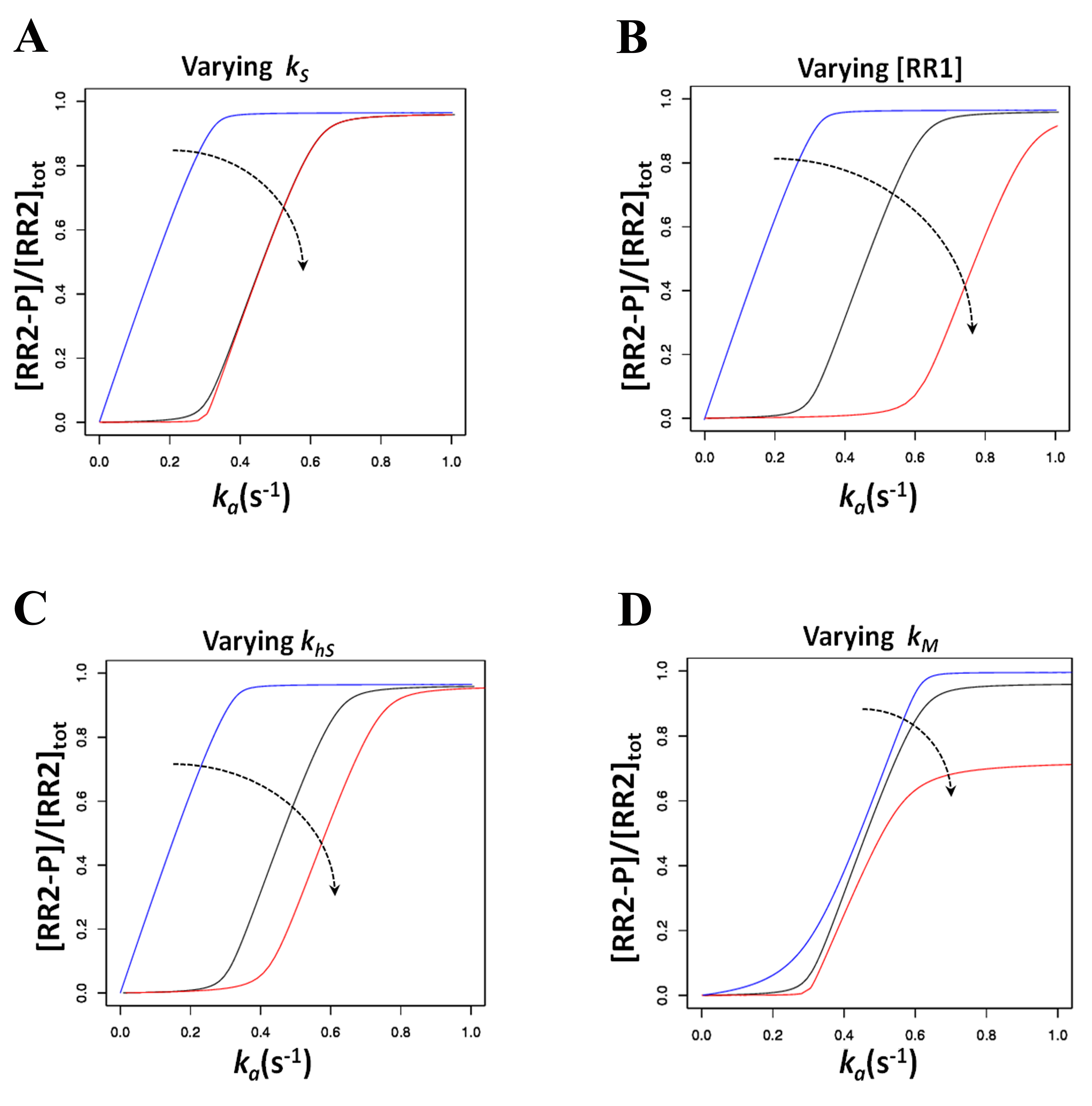

Supplement: Figure S2 — Effect of varying the key parameters in the yeast osmoregulation system on the shape of the signal-response curve. The x- and y-axis show the signal (ka) level and the corresponding level of phosphorylated output RR (SKN7-P) at steady state respectively. Each panel shows a signal-response curve for different parameter values. The results of the basic model are shown in black. The arrow on each panel indicates increasing values of the changed parameter. (A) The forward phosphotransfer rate (kS) for the sink RR was varied from basic model value (of 66.67 µMs−1) to 660, and 0. (B) Concentration of the sink RR was set to 0 µM, 1.5 µM (basic model) and 3 µM. (C) The rate of auto- dephosphorylation of sink RR-P (khS) was set to 0 s−1, 0.5 s−1 (basic model) and 1 s−1. (D) The forward phosphotransfer rate (kM) for the main RR, was set to 1 µMs−1 (basic model), 0.5 µMs−1, and 10 µMs−1. (TIF) [file pcbi.1003890.s002.tif]

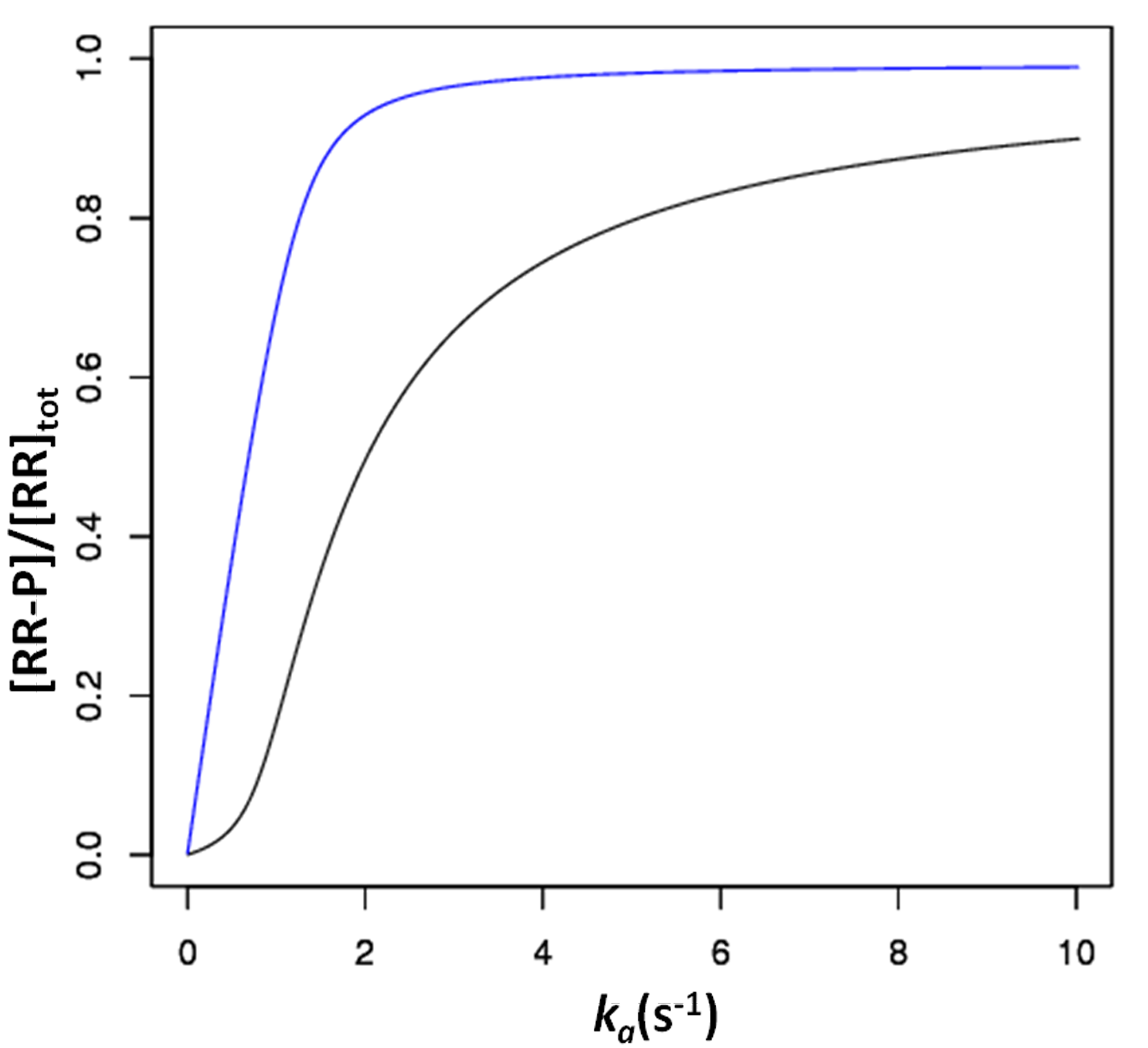

Supplement: Figure S3 — Signal-response relationship for the sink RR and the output RR in the S. meliloti system. The x- and y-axis show the signal (ka) level and the corresponding steady state level of either phosphorylated sink (blue line) or main RR (black line). (TIF) [file pcbi.1003890.s003.tif]

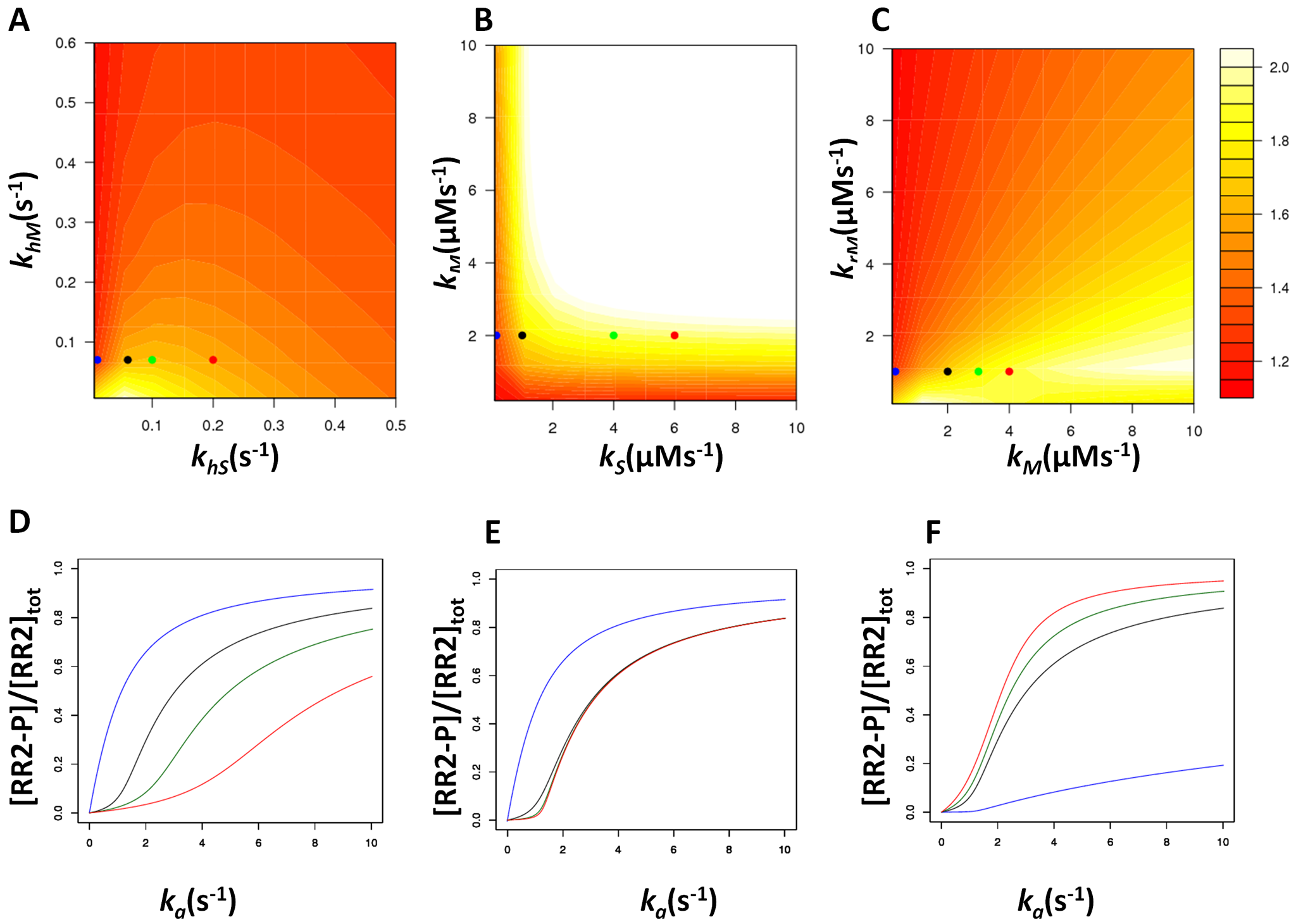

Supplement: Figure S4 — The effect of parameter changes on the signal-response curve of the S. meliloti system. The signal-response curve Hill coefficient is shown on each panel as a heat map. (A) Effect of varying the auto-dephosphorylation rate of the output RR (khM; y-axis) and sink RR (khS; x-axis). (B) Effect of varying the forward phosphotransfer rates to the output and sink RR (kM and kS). (C) Effect of varying the forward and reverse phosphotransfer rates to the output RR (CheY2; x-axis; kM and y-axis; krM). (D–F) Signal-response curves for models corresponding to parameter values indicated as colored circles on the heat maps above; the black circle represents the basic model. (TIF) [file pcbi.1003890.s004.tif]

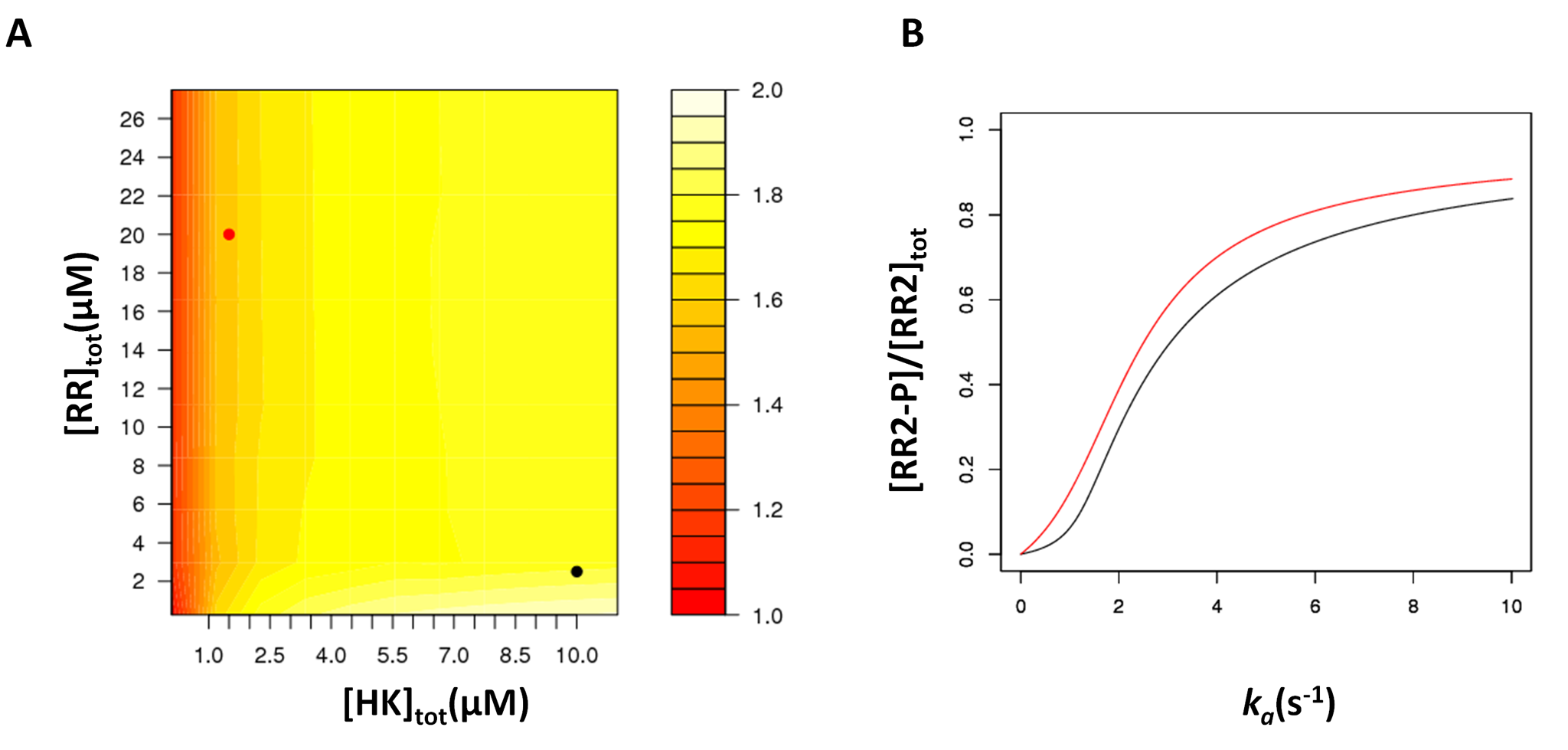

Supplement: Figure S5 — Effect of the stoichiometric ratio of CheA to CheY1, and CheY2 total concentrations on the shape of the signal-response curve for the S. meliloti system. (A) The signal-response curve Hill coefficient is shown as a heat map. The x-axis shows the total concentration of CheA, while the y-axis shows the total concentration of CheY1 and CheY2 (where [CheY1]tot = [CheY2]tot). (B) The signal-response curves resulting from the stoichiometric ratios considered in the in vitro experimental system (10∶2.5∶2.5), in black, and the measured values from S. meliloti (1.5∶20∶20), in red. The corresponding Hill coefficients are 1.75 and 1.59, respectively. (TIF) [file pcbi.1003890.s005.tif]

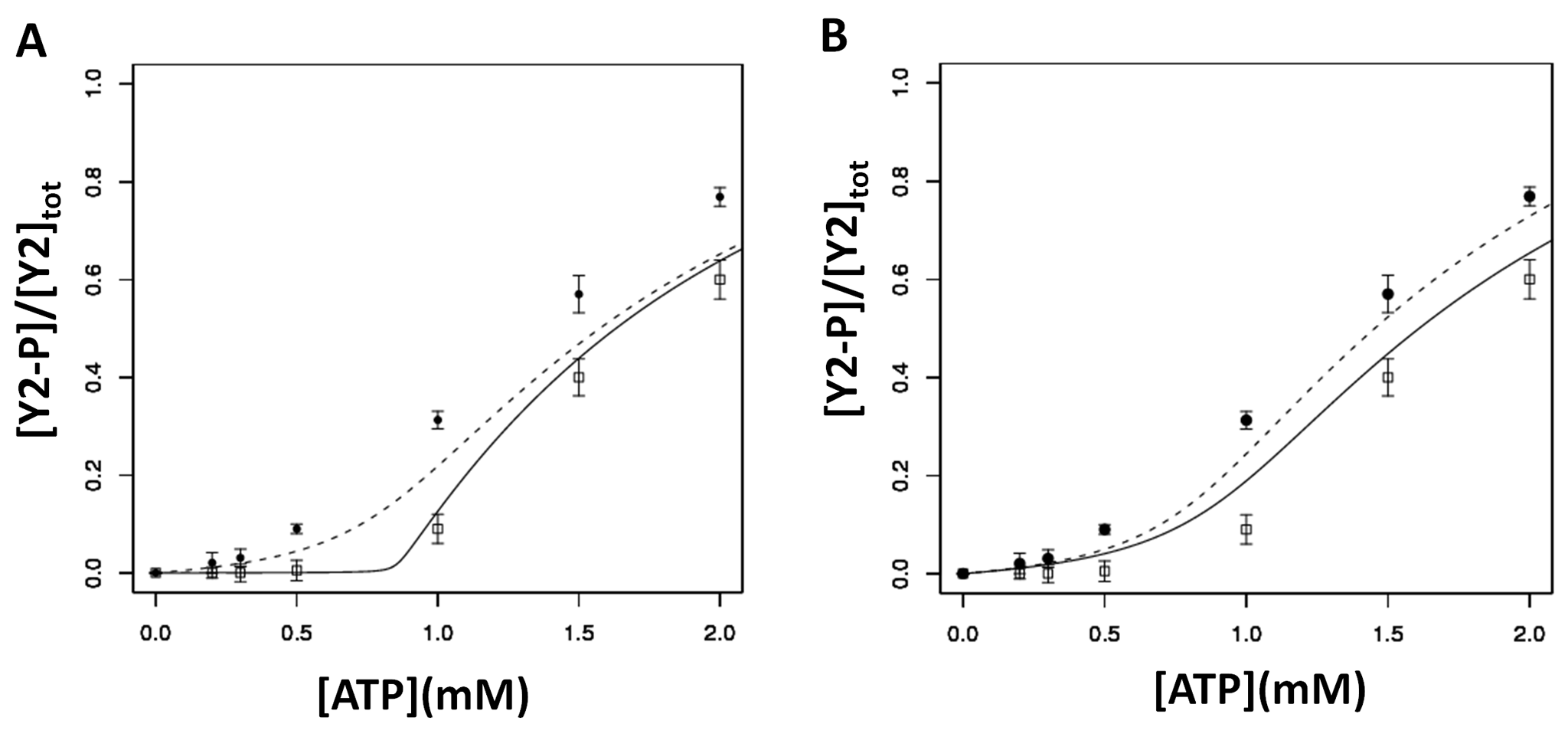

Supplement: Figure S6 — Effect of CheS on the signal-response curve. On each panel, the x- and y-axis show the ATP level and the corresponding steady state phosphorylated CheY2 levels, respectively. The phosphorylated CheY2 levels predicted by the model are shown with a dashed line (absence of CheS) and with a solid line (presence of CheS), while the experimentally measured values are shown in circles and squares on respective graph. Error bars show the standard error of the mean obtained from three independent experiments. Panel A shows the model prediction when only the forward phosphotransfer rate to CheY1 rate is optimized (ks), while panel B shows model prediction when only the CheY1 autodephosphorylation rate (khs) is optimized. (TIF) [file pcbi.1003890.s006.tif]
