# Supplementary material for: Phosphate Sink Containing Two-Component Signaling Systems as Tunable Threshold Devices
Source: PLoS Comput Biol. 2014 Oct 30;10(10):e1003890. doi: 10.1371/journal.pcbi.1003890 (PMC4214558; doi:10.1371/journal.pcbi.1003890)
Supplement: Table S1 — The parameters used for the model of the yeast phosphate sink. (DOC) [file pcbi.1003890.s007.doc]

**Table 1: The parameters used for the model of the yeast phosphate sink**

| Parameter | Description | Value | | Unit | Reference |
| --- | --- | --- | --- | --- | --- |
| *k_a_* | *k*_cat_ for phosphorylation of SLN1 | Varied | | s^-1^ |  |
| *k_C_* | SLN1-P to Receiver domain of SLN-1 Phosphotransfer | 160 | | (µMs) ^-1^ | [45] |
| *k_rC_* | SLN1-P to Receiver domain of SLN-1 Reverse Phosphotransfer | 0 | | (µMs) ^-1^ | [45] |
| *k_T_* | Phosphorylated Receiver domain of SLN-1 to YPD1 Phosphotransfer | 20.7 | | (µMs) ^-1^ | [45] |
| *k_rT_* | Phosphorylated Receiver domain of SLN-1 to YPD1 Reverse Phosphotransfer | 29.5 | | (µMs) ^-1^ | [45] |
| *k_S_* | YPD1-P to SSK1 (sink RR) Phosphotransfer | 66.67 | | (µMs) ^-1^ | [45] |
| *k_rS_* | YPD1-P to SSK1 Reverse phosphotransfer | 0 | | (µMs) ^-1^ | [45] |
| *k_M_* | YPD1-P to SKN7 (main RR) Phosphotransfer | 1 | (µMs) ^-1^ | | [45] |
| *k_rM_* | YPD1-P to SKN7 Reverse phosphotransfer | 0.08 | (µMs) ^-1^ | | [45] |
| *k_hC_* | Autodephosphorylation of Receiver domain of SLN1 | 0.05 | s^-1^ | | [45] |
| *k_hS_* | Autodephosphorylation of SSK1 (sink RR) | 0.05 | s^-1^ | | [45] |
| [SLN]_tot_ | Total conc. of SLN1 | 0.25 | µM | | [45] |
| [Rec domain SLN]_tot_ | Total conc. of Rec domain of SLN1 | 0.25 | µM | | [45] |
| [YPD]_tot_ | Total conc. of YPD | 1.5 | µM | | [45] |
| [SSK1]_tot_ | Total conc. of SSK1 | 1.5 | µM | | [45] |
| [SKN7]_tot_ | Total conc. of SKN7 | 1.5 | µM | | [45] |
