# Supplementary material for: Phosphate Sink Containing Two-Component Signaling Systems as Tunable Threshold Devices
Source: PLoS Comput Biol. 2014 Oct 30;10(10):e1003890. doi: 10.1371/journal.pcbi.1003890 (PMC4214558; doi:10.1371/journal.pcbi.1003890)
Supplement: Text S1 — The main supplementary text. Sections 1 and 2 of this file contain the mathematical analysis of a two-component system with one histidine kinase HK and two response regulators RR. Section 3 describes the mathematical model for the two-component system regulating yeast osmoregulation. (PDF) [file pcbi.1003890.s008.pdf]

Phosphate sink containing two-component signaling systems as tunable threshold devices

Supplementary Text

Munia Amin, Varun Kothamachu, Elisenda Feliu, Birgit E Scharf, Steven L Porter, and Orkun S Soyer

Sections 1 and 2 of this file contain the mathematical analysis of a two-component system with one histidine kinase HK and two response regulators RR. The analytical expression for the signal-response curve is provided, as well as the necessary conditions for sigmoidality. The simple system is described in detail in Section 1. The model that includes complex formation is treated differently and only the second derivative of the signal-response curve at zero is shown (Section 2). Section 3 describes the mathematical model for the two-component system regulating yeast osmoregulation.

List of contents

1. Mathematical analysis of the simple model
2. Mathematical analysis of the model with intermediates
3. A mathematical model for the two-component system regulating yeast osmoregulation
4. Analytical comparison of different models

1 Mathematical analysis of the simple model

1.1 Reactions

The model has 6 species: one histidine kinase HK, two response regulators R1, R2 and their corresponding phosphorylated forms: HKp, R1p, R2p. The first model we analyze consists of the following reactions:

- Auto-phosphorylation of HK:
$$\mathrm{HK} \xrightarrow{k_a} \mathrm{HKp}$$
- Reversible phosphotransfer between HK and R1:
$$\mathrm{HKp} + \mathrm{R1} \xrightarrow{k_S} \mathrm{HK} + \mathrm{R1p} \qquad \mathrm{HK} + \mathrm{R1p} \xrightarrow{k_{rS}} \mathrm{HKp} + \mathrm{R1}$$
- Reversible phosphotransfer between HK and R2:
$$\mathrm{HKp} + \mathrm{R2} \xrightarrow{k_M} \mathrm{HK} + \mathrm{R2p} \qquad \mathrm{HK} + \mathrm{R2p} \xrightarrow{k_{rM}} \mathrm{HKp} + \mathrm{R2}$$
- Auto-dephosphorylation of the response regulators:
$$\mathrm{R1p} \xrightarrow{k_{hS}} \mathrm{R1} \qquad \mathrm{R2p} \xrightarrow{k_{hM}} \mathrm{R2}$$

1.2 System of ODEs

To simplify the notation, we write the concentration of each of the species as:

$$x_1 = [\mathrm{HK}] \qquad x_2 = [\mathrm{HKp}] \qquad x_3 = [\mathrm{R1}] \qquad x_4 = [\mathrm{R1p}] \qquad x_5 = [\mathrm{R2}] \qquad x_6 = [\mathrm{R2p}].$$

We let $x = (x_1, \dots, x_6)$ be the vector of concentrations. We use mass-action kinetics to model the dynamics of the concentrations of the species in time with a system of ordinary differential equations (ODEs). We write the ODE system as the product of the stoichiometric matrix A and the vector of reaction rates. We order the reactions as shown above and the species analogously to the concentrations order $x_1, \dots, x_6$. The stoichiometric matrix A and the vector of reaction rates $v(x)$ are:

$$A = \begin{pmatrix} -1 & 1 & -1 & 1 & -1 & 0 & 0 \\ 1 & -1 & 1 & -1 & 1 & 0 & 0 \\ 0 & -1 & 1 & 0 & 0 & 1 & 0 \\ 0 & 1 & -1 & 0 & 0 & -1 & 0 \\ 0 & 0 & 0 & -1 & 1 & 0 & 1 \\ 0 & 0 & 0 & 1 & -1 & 0 & -1 \end{pmatrix} \qquad v(x) = \begin{pmatrix} k_a x_1 \\ k_S x_2 x_3 \\ k_{rS} x_1 x_4 \\ k_M x_2 x_5 \\ k_{rM} x_1 x_6 \\ k_{hS} x_4 \\ k_{hM} x_6 \end{pmatrix}$$

We let $\dot{x}(t)$ denote $\frac{dx(t)}{dt}$ and drop the dependency on time t in the notation, that is, we write $\dot{x}_i$ and x_i for $\dot{x}_i(t)$ and $x_i(t)$ respectively. The system of ODEs modeling the dynamics of the concentrations in time is

$\dot{x} = Av(x)$, that is:

$$\dot{x}_1 = k_M x_2 x_5 + k_S x_2 x_3 - k_{rM} x_1 x_6 - k_{rS} x_1 x_4 - k_a x_1 \quad \text{(S1)}$$
$$\dot{x}_2 = -k_M x_2 x_5 - k_S x_2 x_3 + k_{rM} x_1 x_6 + k_{rS} x_1 x_4 + k_a x_1 \quad \text{(S2)}$$
$$\dot{x}_3 = -k_S x_2 x_3 + k_{rS} x_1 x_4 + k_{hS} x_4 \quad \text{(S3)}$$
$$\dot{x}_4 = k_S x_2 x_3 - k_{rS} x_1 x_4 - k_{hS} x_4 \quad \text{(S4)}$$
$$\dot{x}_5 = -k_M x_2 x_5 + k_{rM} x_1 x_6 + k_{hM} x_6 \quad \text{(S5)}$$
$$\dot{x}_6 = k_M x_2 x_5 - k_{rM} x_1 x_6 - k_{hM} x_6 \quad \text{(S6)}$$

At steady state, $\dot{x}_i = 0$ for all $i = 1, \ldots, 6$.

The system has three conservation laws:

$$\dot{x}_1 + \dot{x}_2 = 0, \qquad \dot{x}_3 + \dot{x}_4 = 0, \qquad \dot{x}_5 + \dot{x}_6 = 0,$$

which reflect that the sum of species concentration in unphosphorylated and phosphorylated form is constant at each layer. These relations provide three equations at steady state:

$$\overline{H} = x_1 + x_2, \qquad \overline{R}_S = x_3 + x_4, \qquad \overline{R}_M = x_5 + x_6, \quad \text{(S7)}$$

for some positive total amounts $\overline{H}$, $\overline{R}_S$, $\overline{R}_M$. Due to these relations, three of the ODEs, one for each conservation law, are redundant for the computation of the steady states. We choose to disregard (S1), (S3) and (S5).

Therefore, the steady states of the system are the solutions to the steady state equations corresponding to (S2), (S4) and (S6) and the three conservation laws in (S7). That is, the steady states are the solutions to the following six equations:

$$0 = -k_M x_2 x_5 - k_S x_2 x_3 + k_{rM} x_1 x_6 + k_{rS} x_1 x_4 + k_a x_1$$
$$0 = k_S x_2 x_3 - k_{rS} x_1 x_4 - k_{hS} x_4$$
$$0 = k_M x_2 x_5 - k_{rM} x_1 x_6 - k_{hM} x_6$$
$$0 = x_1 + x_2 - \overline{H}$$
$$0 = x_3 + x_4 - \overline{R}_S$$
$$0 = x_5 + x_6 - \overline{R}_M.$$

The equations are easier to analyze if we replace the first equation by the sum of the first three equations. This change does not alter the solutions to the system. The new system to be solved is:

$$0 = k_a x_1 - k_{hS} x_4 - k_{hM} x_6 \quad \text{(S8)}$$
$$0 = k_S x_2 x_3 - k_{rS} x_1 x_4 - k_{hS} x_4 \quad \text{(S9)}$$
$$0 = k_M x_2 x_5 - k_{rM} x_1 x_6 - k_{hM} x_6 \quad \text{(S10)}$$
$$0 = x_1 + x_2 - \overline{H} \quad \text{(S11)}$$
$$0 = x_3 + x_4 - \overline{R}_S \quad \text{(S12)}$$
$$0 = x_5 + x_6 - \overline{R}_M. \quad \text{(S13)}$$

1.3 Expressions for the steady states

We recursively write all concentrations at steady state as a function of x_6. We further impose that all concentrations are positive at steady state.

We **solve for** x_5 at steady state using (S13) and obtain

$$x_5 = \overline{R}_M - x_6. \tag{S14}$$

The right-hand side of this equality decreases in x_6. Further, x_5, x_6 are positive provided that $0 < x_6 < \overline{R}_M$. Let b_1 be this first upper bound for x_6 at a positive steady state, that is

$$b_1 := \overline{R}_M. \tag{S15}$$

We next **solve for** x_1, x_2 using (S11) and (S10) and obtain

$$x_1 = \frac{k_M\overline{H}x_5 - k_{hM}x_6}{k_Mx_5 + k_{rM}x_6}, \qquad x_2 = \frac{x_6\left(k_{rM}\overline{H} + k_{hM}\right)}{k_Mx_5 + k_{rM}x_6}. \tag{S16}$$

At steady state x_2 is positive provided x_5, and x_6 are positive. For x_1 to be positive, we need $k_M\overline{H}x_5 - k_{hM}x_6 > 0$. Substituting x_5 by the expression in (S14), this is equivalent to require that $k_M\overline{H}(\overline{R}_M - x_6) - k_{hM}x_6 > 0$. Isolating x_6 we obtain that the numerator of x_1 is positive if and only if $x_6 < b_2$, where

$$b_2 := \frac{k_M\overline{H}\,\overline{R}_M}{k_M\overline{H} + k_{hM}}. \tag{S17}$$

When $x_6 = b_2$ then $k_M\overline{H}(\overline{R}_M - x_6) - k_{hM}x_6 = 0$ and hence $x_1 = 0$.

Since $\frac{k_M\overline{H}}{k_M\overline{H}+k_{hM}} < 1$, we have that

$$b_2 = \frac{k_M\overline{H}\,\overline{R}_M}{k_M\overline{H} + k_{hM}} < \overline{R}_M = b_1$$

and hence the upper bound b_1 of x_6 in (S15) is larger than b_2 in (S17). Therefore $x_6 < b_1$ is satisfied if also $x_6 < b_2$ and the upper bound for x_6, b_1, can be ignored.

The expression for x_1 in (S16) decreases in x_6 and increases in x_5. Since x_5 decreases in x_6, we conclude that x_1 decreases in x_6. Since $x_2 = \overline{H} - x_1$ (see (S11)), we have that x_2 decreases in x_1 and hence x_2 increases in x_6.

We **solve for** x_3, x_4 using (S9) and (S12) and obtain

$$x_3 = \frac{(k_{rS}x_1 + k_{hS})\overline{R}_S}{k_Sx_2 + k_{rS}x_1 + k_{hS}}, \qquad x_4 = \frac{x_2k_S\overline{R}_S}{k_Sx_2 + k_{rS}x_1 + k_{hS}} \tag{S18}$$

At steady state both x_3, x_4 are positive provided x_1, x_2 are positive. The expression for x_3 decreases in x_2 and increases in x_1. Since x_1 decreases in x_6 and x_2 increases in x_6 we conclude that x_3 decreases in x_6. Since from (S12) we have that $x_3 = \overline{R}_S - x_4$, we conclude that x_4 increases in x_6.

Summary: By iterative substitution, all concentrations at steady state are expressed as functions of x_6. Further, all steady state concentrations are positive if and only if

$$0 < x_6 < \frac{k_M\overline{H}\overline{R}_M}{k_M\overline{H} + k_{hM}}. \tag{S19}$$

1.4 Signal-response curve and maximal response

The value of x_6 at steady state is determined using the only equation not used so far, (S8), after writing all other concentrations as functions of x_6. This equation provides the analytical description of the inverse of the signal-response curve, written as $k_a = f(x_6)$.

Solving (S8) for k_a we obtain

$$k_a = f(x_6) = \frac{k_{hM}x_6 + k_{hS}x_4}{x_1}. \tag{S20}$$

Since x_1 decreases in x_6 and x_4 increases in x_6, the function f is increasing in x_6. For positive steady states, this function needs to be evaluated at the values of x_6 satisfying (S19). When $x_6 = 0$ then $k_a = 0$. As we observed above, when x_6 approaches b_2 we have that x_1 approaches 0, x_2 approaches $\overline{H}$, and hence using (S18) we have that x_4 approaches $\frac{k_S\overline{H}\overline{R}_S}{k_S\overline{H}+k_{hS}}$. Therefore it follows from the expression (S20) that k_a tends to infinity when x_6 tends to the upper bound b_2.

We conclude that for x_6 fulfilling (S19), f is an increasing function with range $(0, +\infty)$. It follows that given a value of signal, $k_a > 0$, then there exists a unique value of x_6 fulfilling (S19) and $k_a = f(x_6)$. This is the steady state value of x_6 for the given input k_a. The concentrations of the other species at steady state are uniquely determined using their expressions as functions of x_6. As a consequence, we showed that multistationarity cannot occur.

The explicit expression of the function $f(x_6)$ in (S20) is:

$$f(x_6) = \frac{x_6 p_1(x_6) p_2(x_6)}{q_1(x_6) q_2(x_6)} \tag{S21}$$

where

$$\begin{aligned}
q_1(x_6) &= k_M(k_{rS}\overline{H} + k_{hS})(\overline{R}_M - x_6) + k_{rM}x_6(k_S\overline{H} + k_{hS}) + k_{hM}x_6(k_S - k_{rS}) \\
q_2(x_6) &= k_M\overline{H}(\overline{R}_M - x_6) - k_{hM}x_6 \\
p_1(x_6) &= \overline{R}_S k_S k_{hS}(k_{rM}\overline{H} + k_{hM}) + k_{hM}q_1(x_6) \\
p_2(x_6) &= k_M(\overline{R}_M - x_6) + k_{rM}x_6.
\end{aligned}$$

The signal-response curve is the inverse of the function f. Since the function $f(x_6)$ increases, so does the signal-response curve. Further, we conclude that the upper bound b_2 is the level of phosphorylated R2 that the system attains when k_a increases infinitely. In other words,

$$r_{max} = \frac{k_M\overline{H}\,\overline{R}_M}{k_M\overline{H} + k_{hM}} \tag{S22}$$

is the maximal level of response of the system. The maximal response levels for HK and R1 are $\overline{H}$ and $\frac{k_S\overline{H}\,\overline{R}_S}{k_S\overline{H}+k_{hS}}$ respectively.

Observe that the maximal response (S22) does not depend on any of the rate constants or total amounts involving the sink-RR (R1). Therefore, the presence of a sink does not alter the maximal response, but might alter the shape of the signal-response curve. More generally, the inverse of the signal-response curve without the sink is:

$$f(x_6) = \frac{(k_M(\overline{R}_M - x_6) + k_{rM}x_6)k_{hM}x_6}{k_M\overline{H}(\overline{R}_M - x_6) - k_{hM}x_6} = \frac{k_{hM}x_6 p_2(x_6)}{q_2(x_6)}. \tag{S23}$$

This is obtained by showing as above that in this case

$$k_a = \frac{k_{hM} x_6}{x_1}. \tag{S24}$$

Alternatively, we can set $\overline{R}_S = 0$ and all rate constants of the sink system to zero in the expression of f in (S21). Note that the expression of x_1 in (S16) does not depend on the presence of sink. Therefore, for any fixed x_6, (S24) and (S20) satisfy the inequality

$$\frac{k_{hM} x_6}{x_1} < \frac{k_{hM} x_6 + k_{hS} x_4}{x_1}$$

after substituting x_1, x_4 for their expressions in x_6 and for any choice of rate constants of the sink system and $\overline{R}_S$. We deduce that given a response x_6, k_a in (S20) is larger than k_a in (S24). In other words, presence of the sink causes the system to require more signal to achieve the same level of response. If we plot the signal-response curve for a system with a sink, then the graph is below the signal-response curve of the corresponding system without sink, while keeping the common rates the same.

1.5 First derivative at zero

The first derivative of the signal-response curve at zero is computed as $1/f'(0)$ and takes the value:

$$\frac{k_M(k_{rS}\overline{H} + k_{hS})\overline{H}\overline{R}_M}{k_S k_{hS}(k_{rM}\overline{H} + k_{hM})\overline{R}_S + k_M k_{hM}(k_{rS}\overline{H} + k_{hS})\overline{R}_M}. \tag{S25}$$

The signal-response curve of the corresponding system without sink has first derivative at zero:

$$\frac{\overline{H}}{k_{hM}}.$$

Observe that (S25) can be rewritten as

$$\frac{k_M(k_{rS}\overline{H} + k_{hS})\overline{R}_M}{\frac{k_S k_{hS}(k_{rM}\overline{H}+k_{hM})\overline{R}_S}{k_{hM}} + k_M(k_{rS}\overline{H} + k_{hS})\overline{R}_M} \cdot \frac{\overline{H}}{k_{hM}},$$

and it becomes apparent that the first derivative at zero of the signal-response curve when the system has a sink is always smaller than the corresponding first derivative at zero of the signal-response curve of the system without a sign. Therefore, presence of the sink reduces the slope of the curve around zero.

1.6 Sigmoidal vs. hyperbolic

A hyperbolic curve has negative second derivative in all its domain, while a sigmoidal curve displays a change of sign of the second derivative. In particular, the second derivative at zero must be positive for a sigmoidal curve and negative for a hyperbolic curve.

The second derivative at zero of the signal-response curve can be computed from its inverse, f. In particular, the sign of the second derivative of the signal-response curve at zero is minus the sign of $f''(0)$ and is given by the sign of:

$$S = -k_S k_{hS}\overline{R}_S(\omega_1\overline{H}(k_{rS} - k_S) + \omega_2(k_M\overline{H} + k_{hM})) - k_M k_{hM}\omega_2^2\overline{R}_M \tag{S26}$$

where

$$\omega_1 = k_{rM}\overline{H} + k_{hM} \qquad \omega_2 = k_{rS}\overline{H} + k_{hS}.$$

For S to be positive, we necessarily need that

$$k_S k_{hS} \overline{R}_S \neq 0 \qquad \text{and} \qquad k_{rS} < k_S$$

as stated in the main text. These conditions are necessary for sigmoidality. In particular, presence of the sink is necessary. A simple system containing only one HK and one response regulator can only display hyperbolic signal-response curves.

The above conditions are necessary for sigmoidality. Sufficient conditions can also be given. For example, if

$$\frac{k_{rS}}{k_S} < \frac{k_{rM}}{k_M + k_{rM}}$$

then there exist total amounts $\overline{H}, \overline{R}_S$ large enough and $\overline{R}_M$ small enough such that the signal-response curve is sigmoidal. This follows from a general fact on polynomials. Consider a polynomial $p(x) = a_n x^n + \cdots + a_1 x + a_0$. If $a_n > 0$, then for x_0 large enough, $p(x_0) > 0$. Similarly, if $a_n < 0$, then for x_0 large enough, $p(x_0) < 0$. We consider the term S in (S26) as a polynomial in $\overline{H}$. This polynomial has degree 2 and the coefficient of the term of degree 2 is

$$-k_S k_{hS}(k_{rS}k_M - k_{rM}k_S + k_{rM}k_{rS})\overline{R}_S - k_{rS}^2 k_{hM} k_M \overline{R}_M.$$

If $k_{rS}k_M - k_{rM}k_S + k_{rM}k_{rS} < 0$ or, equivalently, $\frac{k_{rS}}{k_S} < \frac{k_{rM}}{k_M + k_{rM}}$, then for $\overline{R}_S$ large and $\overline{R}_M$ small the coefficient of the polynomial is positive and we can use the general fact on polynomials to conclude that for $\overline{H}$ large enough, S is positive and hence sigmoidality occurs.

2 Mathematical analysis of the model with intermediates

We consider here the case where the model includes complex formation in the phosphotransfer reactions.

2.1 Reactions

The model has now 8 species: one histidine kinase HK, two response regulators R1, R2, their corresponding phosphorylated forms, HKp, R1p, R2p, and two intermediates Y_S, Y_M. The model consists of the following reactions:

- Auto-phosphorylation of HK:

$$\text{HK} \xrightarrow{k_a} \text{HKp}$$

- Reversible phosphotransfer between HK and R1 through the formation of a complex:

$$\text{HKp} + \text{R1} \xrightarrow{k_{aS}} Y_S \xrightarrow{k_{bS}} \text{HK} + \text{R1p} \qquad \text{HK} + \text{R1p} \xrightarrow{k_{brS}} Y_S \xrightarrow{k_{arS}} \text{HKp} + \text{R1}$$

- Reversible phosphotransfer between HK and R2:

$$\text{HKp} + \text{R2} \xrightarrow{k_{aM}} Y_M \xrightarrow{k_{bM}} \text{HK} + \text{R2p} \qquad \text{HK} + \text{R2p} \xrightarrow{k_{brM}} Y_M \xrightarrow{k_{arM}} \text{HKp} + \text{R2}$$

- Auto-dephosphorylation of the response regulators:

$$\text{R1p} \xrightarrow{k_{hS}} \text{R1} \qquad \text{R2p} \xrightarrow{k_{hM}} \text{R2}$$

2.2 System of ODEs

To simplify the notation, we write the concentrations of each of the species as:

$$x_1 = [\text{HK}] \quad x_2 = [\text{HKp}] \quad x_3 = [\text{R1}] \quad x_4 = [\text{R1p}] \quad x_5 = [\text{R2}] \quad x_6 = [\text{R2p}] \quad x_7 = [Y_S] \quad x_8 = [Y_M].$$

We let $x = (x_1, \dots, x_8)$ be the vector of concentrations. We proceed as above to construct a system of ODEs for this model:

$$\dot{x}_1 = -k_{brM}x_1x_6 - k_{brS}x_1x_4 - k_a x_1 + k_{bM}x_8 + k_{bS}x_7 \tag{S27}$$
$$\dot{x}_2 = -k_{aM}x_2x_5 - k_{aS}x_2x_3 + k_a x_1 + k_{arM}x_8 + k_{arS}x_7 \tag{S28}$$
$$\dot{x}_3 = -k_{aS}x_2x_3 + k_{arS}x_7 + k_{hS}x_4 \tag{S29}$$
$$\dot{x}_4 = -k_{brS}x_1x_4 + k_{bS}x_7 - k_{hS}x_4 \tag{S30}$$
$$\dot{x}_5 = -k_{aM}x_2x_5 + k_{arM}x_8 + k_{hM}x_6 \tag{S31}$$
$$\dot{x}_6 = -k_{brM}x_1x_6 + k_{bM}x_8 - k_{hM}x_6 \tag{S32}$$
$$\dot{x}_7 = k_{aS}x_2x_3 + k_{brS}x_1x_4 - k_{arS}x_7 - k_{bS}x_7 \tag{S33}$$
$$\dot{x}_8 = k_{aM}x_2x_5 + k_{brM}x_1x_6 - k_{arM}x_8 - k_{bM}x_8. \tag{S34}$$

At steady state, $\dot{x}_i = 0$ for all $i = 1, \dots, 8$. The system has also three conservation laws:

$$\dot{x}_1 + \dot{x}_2 + \dot{x}_7 + \dot{x}_8 = 0, \qquad \dot{x}_3 + \dot{x}_4 + \dot{x}_7 = 0, \qquad \dot{x}_5 + \dot{x}_6 + \dot{x}_8 = 0,$$

which provide three equations at steady state:

$$\overline{H} = x_1 + x_2 + x_7 + x_8, \qquad \overline{R}_S = x_3 + x_4 + x_7, \qquad \overline{R}_M = x_5 + x_6 + x_8 \tag{S35}$$

for positive total amounts $\overline{H}, \overline{R}_S$ and $\overline{R}_M$. Three of the steady state equations are redundant and are substituted by the conservation law equations. We keep the steady state equations corresponding to (S28), (S30), (S32), (S33) and (S34). Therefore, the steady states constrained to the conservation laws are given as the solutions to the equations:

$$\begin{aligned}
0 &= -k_{aM}x_2x_5 - k_{aS}x_2x_3 + k_a x_1 + k_{arM}x_8 + k_{arS}x_7 \\
0 &= -k_{brS}x_1x_4 + k_{bS}x_7 - k_{hS}x_4 \\
0 &= -k_{brM}x_1x_6 + k_{bM}x_8 - k_{hM}x_6 \\
0 &= k_{aS}x_2x_3 + k_{brS}x_1x_4 - k_{arS}x_7 - k_{bS}x_7 \\
0 &= k_{aM}x_2x_5 + k_{brM}x_1x_6 - k_{arM}x_8 - k_{bM}x_8 \\
0 &= x_1 + x_2 + x_7 + x_8 - \overline{H} \\
0 &= x_3 + x_4 + x_7 - \overline{R}_S \\
0 &= x_5 + x_6 + x_8 - \overline{R}_M.
\end{aligned} \tag{S36}$$

2.3 Sigmoidal vs. hyperbolic

We compute directly the second derivative of the signal-response curve at zero. First of all, we observe that when the signal is zero ($k_a = 0$), the steady state is:

$$x_1 = \overline{H}, \quad x_3 = \overline{R}_S, \quad x_5 = \overline{R}_M, \quad x_2 = x_4 = x_6 = x_7 = x_8 = 0.$$

We differentiate each equation in (S36) with respect to k_a. To simplify the notation, we write

$$p_i = \frac{dx_i(k_a)}{dk_a}$$

and evaluate the concentrations x_i at $k_a = 0$. We obtain a new system of equations:

$$\begin{aligned}
0 &= -k_{aS}\overline{R}_S\, p_2 - \overline{R}_M k_{aM}\, p_2 + k_{arM}\, p_8 + k_{arS}\, p_7 + \overline{H} \\
0 &= -k_{brS}\overline{H}\, p_4 + k_{bS}\, p_7 - k_{hS}\, p_4 \\
0 &= -k_{brM}\overline{H}\, p_6 + k_{bM}\, p_8 - k_{hM}\, p_6 \\
0 &= k_{brS}\overline{H}\, p_4 + k_{aS}\overline{R}_S\, p_2 - k_{arS}\, p_7 - k_{bS}\, p_7 \\
0 &= k_{brM}\overline{H}\, p_6 + k_{aM}\overline{R}_M\, p_2 - k_{arM}\, p_8 - k_{bM}\, p_8 \\
0 &= p_1 + p_2 + p_7 + p_8 \\
0 &= p_3 + p_4 + p_7 \\
0 &= p_5 + p_6 + p_8.
\end{aligned} \tag{S37}$$

The solutions in p_i are the derivatives at zero of each of the concentrations at steady state as a function of k_a. For instance, the **first derivative** of x_6, that is, of the signal-response curve, at zero is

$$\frac{k_{aM}k_{bM}\left(k_{arS}k_{brS}\overline{H} + (k_{arS} + k_{bS})k_{hS}\right)\overline{H}\,\overline{R}_M}{k_{aS}k_{bS}k_{hS}(k_{arM}k_{brM}\overline{H} + (k_{bS} + k_{bM})k_{hM})\overline{R}_S + k_{aM}k_{hM}k_{bM}(k_{arS}k_{brS}\overline{H} + (k_{arS} + k_{bS})k_{hS})\overline{R}_M}.$$

We introduce new rate constants that allow us to compare the systems with and without intermediates. That is, we define the inverse of the Michaelis-Menten constants of the intermediates:

$$k_{yS} = \frac{k_{aS}}{k_{arS} + k_{bS}}, \quad k_{yM} = \frac{k_{aM}}{k_{arM} + k_{bM}}, \quad k_{yrS} = \frac{k_{brS}}{k_{arS} + k_{bS}}, \quad k_{yrM} = \frac{k_{brM}}{k_{arM} + k_{bM}}$$

and let the new effective reaction rate constants for the phosphotransfer reactions be:

$$k_S = k_{bS}k_{yS}, \quad k_M = k_{bM}k_{yM}, \quad k_{rS} = k_{arS}k_{yrS}, \quad k_{rM} = k_{arM}k_{yrM}.$$

With the new constants, the first derivative at zero of the signal-response curve is

$$\frac{k_M(k_{rS}\overline{H} + k_{hS})\overline{R}_M\overline{H}}{k_S k_{hS}(k_{rM}\overline{H} + k_{hM})\overline{R}_S + k_M k_{hM}(k_{rS}\overline{H} + k_{hS})\overline{R}_M},$$

which is identical to the first derivative of the signal-response curve at zero without modeling of intermediates (given in (S25)). That is, with the identification of constants, the two derivatives are identical.

We compute now the **second derivative** of each variable at zero ($k_a = 0$) following the same procedure as above. We differentiate the equations (S37), evaluate the concentrations x_i at $k_a = 0$ and the first derivatives p_i at the solutions obtained in the first step. We obtain a new system of equations on the second derivatives of x_i with respect to k_a at zero and solve it. In particular, we obtain the second derivative of x_6 with respect to k_a at zero and conclude that the sign of the second derivative of the signal-response curve at zero agrees with the sign of the following term:

$$\begin{aligned}
S_y = \omega_1\omega_2 S &- ((k_{yrM}k_M\overline{H} + k_{yM}\omega_1)\omega_2 - (k_{yrS}k_S\overline{H} + k_{yS}\omega_2)\omega_1)k_S k_{hS}\overline{H}\overline{R}_S\omega_1\omega_2 \\
&- ((k_{yrM}k_M\overline{H} + k_{yM}\omega_1)\omega_2\overline{R}_M + (k_{yrS}k_S\overline{H} + k_{yS}\omega_2)\omega_1\overline{R}_S)(k_M k_{hM}\omega_2^2\overline{R}_M \\
&+ (k_{rS}\omega_1\overline{H} + k_{hM}\omega_2)k_S k_{hS}\overline{R}_S)
\end{aligned} \tag{S38}$$

where

$$\omega_1 = k_{rM}\overline{H} + k_{hM} \qquad \omega_2 = k_{rS}\overline{H} + k_{hS}$$

and S corresponds to the system without intermediates, and was given in (S26) to be

$$S = -k_S k_{hS}(\omega_1 \overline{H}(k_{rS} - k_S) + \omega_2(k_M \overline{H} + k_{hM}))\overline{R}_S - k_M k_{hM} \omega_2^2 \overline{R}_M.$$

Observe that the terms not involving the Michaelis-Menten constants of the intermediates correspond precisely to the sigmoidality term S without intermediates.

For S_y to be positive one needs either S in (S26) to be positive or the blue term to be negative. The latter involves the Michaelis-Menten constants of the intermediates and, in particular, the condition $k_{rS} < k_S$ is not necessary anymore. What is still necessary is that

$$k_S k_{hS} \overline{R}_S \neq 0.$$

Let us look closely at the extra term that can be negative. Sigmoidality cannot occur if $k_{rS} > k_S$ and

$$(k_M k_{yrM} \overline{H} + k_{yM}\omega_1)\omega_2 - (k_S k_{yrS}\overline{H} + k_{yS}\omega_2)\omega_1 > 0.$$

The left-hand side of the inequality is written as a polynomial in $\overline{H}$ as

$$\begin{aligned} S_y &= (k_M k_{rS} k_{yrM} + k_{rM}(k_{rS}(k_{yM} - k_{yS}) - k_S k_{yrS}))\,\overline{H}^2 \\ &\quad + (k_M k_{hS} k_{yrM} + k_{hM}(k_{rS}(k_{yM} - k_{yS}) - k_S k_{yrS}) + k_{hS} k_{rM}(k_{yM} - k_{yS}))\,\overline{H} + k_{hM} k_{hS}(k_{yM} - k_{yS}). \end{aligned}$$

All the coefficients of this polynomial are positive if $k_{rS}(k_{yM} - k_{yS}) - k_S k_{yrS} > 0$, that is, if

$$\frac{k_S}{k_{rS}} < \frac{k_{yM} - k_{yS}}{k_{yrS}}. \tag{S39}$$

Hence sigmoidality cannot occur if $k_{rS} > k_S$ and (S39) holds. We conclude that necessary conditions for sigmoidality in the model with intermediates are that

(i) $k_S k_{hS} \overline{R}_S \neq 0$, and

(ii) $\frac{k_S}{k_{rS}} > \min\left(1, \frac{k_{yM} - k_{yS}}{k_{yrS}}\right)$.

In particular, if $k_{yM} < k_{yS}$, then we are left with the same necessary conditions as in the case without intermediates. In terms of the original reaction rate constants, we note that

$$\frac{k_S k_{yrS} + k_{rS} k_{yS}}{k_{rS}} = \frac{k_{aS}}{k_{arS}} \qquad \text{and} \qquad \frac{k_S}{k_{rS}} = \frac{k_{aS} k_{bS}}{k_{arS} k_{brS}}. \tag{S40}$$

Then necessary conditions for sigmoidality are that

(i) $k_S k_{hS} \overline{R}_S \neq 0$, and

(ii) $\frac{k_{aS}}{k_{arS}} > \min\left(\frac{k_{brS}}{k_{bS}}, \frac{k_{aM}}{k_{arM} + k_{bM}}\right)$.

Finally, we look for conditions that guarantee the existence of sigmoidality for some total amounts. To this end we proceed as above and consider S_y as a polynomial in $\overline{H}$. The polynomial has degree five and the coefficient of highest degree is:

$$-\overline{R}_S k_S k_{hS} k_{rM} k_{rS} \left(k_M k_{rS} k_{yrM} - k_S k_{rM} k_{yrS} + k_{rM} k_{rS}(k_{yM} - k_{yS})\right). \tag{S41}$$

If the coefficient in is positive, then for $\overline{H}$ large enough, S_y is positive and as a consequence the signal-response curve is sigmoidal. The coefficient is positive if and only if

$$(k_M k_{yrM} + k_{rM} k_{yM}) k_{rS} < (k_S k_{yrS} + k_{rS} k_{yS}) k_{rM},$$

or equivalently (cf. (S40)), if and only if

$$\frac{k_{aM}}{k_{arM}} < \frac{k_{aS}}{k_{arS}}.$$

3 A mathematical model for the two-component system regulating yeast osmoregulation

To model the one HK-two RR system found in yeast osmoregulation, we considered its dynamics in isolation of other cellular components. In this system, the HK (SLN1) is a hybrid protein composed of histidine kinase (HK) and receiver (RC) domains and there exists an additional histidine phosphotransfer (HPT) protein (YPD1). Together, these constitute a phosphorelay, where the YPD1 phosphotranfers to the two RRs, SSK1 and SKN7. We have modeled the hybrid HK as two separate proteins. The reactions in this system are:

$$\begin{aligned}
\text{HK} &\xrightarrow{k_a} \text{HKp} \\
\text{RC} + \text{HKp} &\underset{k_{rC}}{\overset{k_C}{\rightleftharpoons}} \text{RCp} + \text{HK} \\
\text{HPT} + \text{RCp} &\underset{k_{rT}}{\overset{k_T}{\rightleftharpoons}} \text{HPTp} + \text{RC} \\
\text{RR1} + \text{HPTp} &\underset{k_{rS}}{\overset{k_S}{\rightleftharpoons}} \text{RR1p} + \text{HPT} \\
\text{RR2} + \text{HPTp} &\underset{k_{rM}}{\overset{k_M}{\rightleftharpoons}} \text{RR2p} + \text{HPT} \\
\text{RCp} &\xrightarrow{k_{hC}} \text{RC} + \text{Pi} \\
\text{RR1p} &\xrightarrow{k_{hS}} \text{RR1} + \text{Pi} \\
\text{RR2p} &\xrightarrow{k_{hM}} \text{RR2} + \text{Pi}
\end{aligned}$$

where HK, RC, HPT, RR1 and RR2 stand for SLN1, its receiver domain, YPD1, SSK1, and SKN7 respectively and the -p suffix represents phosphorylated forms of these proteins/domains. The above reaction scheme can be used to derive a system of ordinary differential equations, which describe the changes in concentrations over time. For the phosphorylated forms, the system is:

$$\frac{d[\text{HKp}]}{dt} = k_a[\text{HK}] + k_{rC}[\text{RCp}][\text{HK}] - k_C[\text{RC}][\text{HKp}]$$

$$\frac{d[\text{RCp}]}{dt} = k_C[\text{RC}][\text{HKp}] + k_{rT}[\text{HPTp}][\text{RC}] - k_{rC}[\text{RCp}][\text{HK}] - k_T[\text{HPT}][\text{RCp}] - k_{hC}[\text{RCp}]$$

$$\frac{d[\text{HPTp}]}{dt} = k_T[\text{HPT}][\text{RCp}] + k_{rS}[\text{RR1p}][\text{HPT}] + k_{rM}[\text{RR2p}][\text{HPT}] - k_{rT}[\text{HPTp}][\text{RC}] - k_S[\text{RR1}][\text{HPTp}] - k_M[\text{RR2}][\text{HPTp}]$$

$$\frac{d[\text{RR1p}]}{dt} = k_S[\text{RR1}][\text{HPTp}] - k_{rS}[\text{RR1p}][\text{HPT}] - k_{hS}[\text{RR1p}]$$

$$\frac{d[\text{RR2p}]}{dt} = k_M[\text{RR2}][\text{HPTp}] - k_{rM}[\text{RR2p}][\text{HPT}] - k_{hM}[\text{RR2p}].$$

In addition, we have five conservation equations:

$$[\text{HK}]_{\text{tot}} = [\text{HK}] + [\text{HKp}]$$
$$[\text{RC}]_{\text{tot}} = [\text{RC}] + [\text{RCp}]$$
$$[\text{HPT}]_{\text{tot}} = [\text{HPT}] + [\text{HPTp}]$$
$$[\text{RR1}]_{\text{tot}} = [\text{RR1}] + [\text{RR1p}]$$
$$[\text{RR2}]_{\text{tot}} = [\text{RR2}] + [\text{RR2p}].$$

To analyze the behavior of the phosphate sink motif with increasing signal, we simulated the incoming signals from receptors as an increase in the auto-phosphorylation constant rate of the kinase (k_a). The model was parameterized with data from literature (see Supplementary Table 1). We numerically integrated the model to derive steady state signal-response relationships. The latter analysis gives the steady state level of phosphorylated RR2 at a given signal (k_a), where signal was taken as the constant rate of auto-phosphorylation of kinase and allows deriving a so-called signal-response curve. This curve is found by numerically integrating the system to steady state at a fixed signal level and then numerically "following" this steady state (i.e. steady state level of phosphorylated RR2), while changing the signal. This analysis is equivalent to allowing the system to reach steady state under different signal values. Both signal-response analyses were performed using the software Oscill8 (http://oscill8.sourceforge.net/).

4 Analytical comparison of different models

To perform a formal check for the potential of bistability in the different models (discussed in the main text), we have utilized the chemical network theory. This theory provides several analytical tests that can provide a definite answer on the possibility of existence of multiple stationary states in a given reaction network. We have applied these tests to the basic model and model with alternative reaction schemes; we had devised using the Chemical Network Tool v2.2 (http://www.chbmeng.ohio-state.edu/ feinberg/crntwin/). The model files used with this tool and describing the chemical reaction systems, as well as the analytical results from the tool are provided as separate files. Supplementary Text 2 contains the reaction system described in the basic model without complex formation. Supplementary Text 3 contains the reaction system with formation of complexes during phosphotransfer reactions. The four complexes between the phosphorylated/unphosphorylated CheA and the phosphorylated/unphosphorylated CheY1 and CheY2. In these reaction systems A, Y1 and Y2 stand for CheA, CheY1 and CheY2 respectively. P refers to phosphorylated form.
