# Supplementary material for: Phosphate Sink Containing Two-Component Signaling Systems as Tunable Threshold Devices
Source: PLoS Comput Biol. 2014 Oct 30;10(10):e1003890. doi: 10.1371/journal.pcbi.1003890 (PMC4214558; doi:10.1371/journal.pcbi.1003890)
Supplement: Text S3 — This file contains the results of the analysis using the Chemical Reaction Network toolbox for the reaction system with formation of complexes during phosphotransfer reactions. (DOC) [file pcbi.1003890.s010.doc]

**Phosphate sink containing two-component signaling systems as tunable threshold devices**

**Munia Amin, Varun Kothamachu, Elisenda Feliu, Birgit E Scharf, Steven L Porter, and Orkun S Soyer**

**Supplementary Text 3**

This file contains the reaction system with formation of complexes during phosphotransfer reactions.

BASIC REPORT: NoName1

=====================

Reaction network:

¯¯¯¯¯¯¯¯¯¯¯¯¯¯¯¯

A -> AP

A + Y1 <-> AY1

AY1 <-> APY1

APY1 <-> A + Y1P

APY1 <-> AP + Y1

AP + Y2 <-> APY2

APY2 <-> A + Y2P

APY2 <-> AY2

AY2 <-> A + Y2

Y1P -> Y1

Y2P -> Y2

Remark: None.

¯¯¯¯¯¯

Graphical Properties

====================

Number of complexes = 16

Number of linkage classes = 5:

Linkage class no. 1: {A, AP}

Linkage class no. 2: {A + Y1, AY1, APY1, A + Y1P, AP + Y1}

Linkage class no. 3: {AP + Y2, APY2, A + Y2P, AY2, A + Y2}

Linkage class no. 4: {Y1P, Y1}

Linkage class no. 5: {Y2P, Y2}

Number of TERMINAL strong linkage classes = 5:

Strong linkage class no. 1: {A + Y1, AY1, APY1, A + Y1P, AP + Y1}

Strong linkage class no. 2: {AP + Y2, APY2, A + Y2P, AY2, A + Y2}

Strong linkage class no. 3: {AP}

Strong linkage class no. 4: {Y1}

Strong linkage class no. 5: {Y2}

Number of NON-TERMINAL strong linkage classes = 3:

Strong linkage class no. 6: {A}

Strong linkage class no. 7: {Y1P}

Strong linkage class no. 8: {Y2P}

The network is neither reversible nor weakly reversible.

Rank Information

================

Rank of entire network = 7

Deficiency Information

======================

Deficiency of entire network = 4

Deficiency of linkage class no. 1 = 0

Deficiency of linkage class no. 2 = 0

Deficiency of linkage class no. 3 = 0

Deficiency of linkage class no. 4 = 0

Deficiency of linkage class no. 5 = 0

Analysis

========

This is a deficiency four network. It is a good candidate for application

of HIGHER DEFICIENCY THEORY (tailored mostly to networks with deficien-

cies greater than one).

Whether results will be obtained, will depend on whether or not the reaction

network has certain additional structural attributes that help reduce the problem

to a study of systems of linear inequalities.

If a network is "good", higher deficiency theory will determine, either

affirmatively or negatively, whether there are positive rate constant values

such that the corresponding mass action differential equations admit multiple

(positive) steady states. If the answer is affirmative, higher deficiency

theory will generate a sample set of rate constants and a pair of distinct

steady states that are consistent with those rate constants.

If a network is "bad", some additional nonlinear analysis might be required,

and the program might not be able to ascertain the network's capacity for

multiple positive steady states. If definite conclusions can be reached they

they will be reported. Otherwise the program will tell you that it cannot reach

a conclusion.

Higher deficiency theory will also determine, either affirmatively or

negatively, whether there can exist a set of rate constants such that the

corresponding mass action differential equations admit a positive steady

state having a zero eigenvalue (corresponding to an eigenvector in the

stoichiometric subspace). When the answer is affirmative, the theory will

produce such a set of rate constants, a positive steady state, and an

eigenvector (in the stoichiometric subspace) corresponding to an eigenvalue

of zero. Results of this kind are contained after running the Zero Eigenvalue

Report.

=================================

HIGHER DEFICIENCY REPORT: NoName1

=================================

Analysis

========

Taken with mass action kinetics, the network DOES have the capacity for

multiple steady states. That is, there are rate constants that give rise to

two or more positive (stoichiometrically compatible) steady states --

you'll see an example below -- and also rate constants for which there is a

steady state having an eigenvector (in the stoichiometric subspace)

corresponding to an eigenvalue of zero. (To construct rate constants that

give a degenerate steady state, use the Zero Eigenvalue Report.)

A mass action system example is also given below:

Example No. 1: Multiple Steady States

¯¯¯¯¯¯¯¯¯¯¯¯¯¯¯¯¯¯¯¯¯¯¯¯¯¯¯¯¯¯¯¯¯¯¯¯¯

The following mass action system gives rise to multiple steady states:

A ---53.264249-> AP

A + Y1 ---525.88864-> AY1

AY1 ---76.689969-> A + Y1

AY1 ---7356.1463-> APY1

APY1 ---6224.388--> AY1

APY1 ---7843.4039-> A + Y1P

APY1 ---1043.064--> AP + Y1

A + Y1P ---20534.231-> APY1

AP + Y1 ---132.87987-> APY1

AP + Y2 ---116491.28-> APY2

APY2 ---4951.8227-> AP + Y2

APY2 ---8571.821--> A + Y2P

APY2 ---140.24927-> AY2

A + Y2P ---5955.1979-> APY2

AY2 -------1-----> APY2

AY2 ---32.105055-> A + Y2

A + Y2 ---11290.218-> AY2

Y1P ---424.3858--> Y1

Y2P ---123.07748-> Y2

The steady states shown below are both consistent with the mass

action system indicated.

Steady State No. 1 Species Steady State No. 2

¯¯¯¯¯¯¯¯¯¯¯¯¯¯¯¯¯¯ ¯¯¯¯¯¯¯ ¯¯¯¯¯¯¯¯¯¯¯¯¯¯¯¯¯¯

8.8663 E-2 A 0.17854626

1.2542 E-2 AP 0.10242547

2.8335553 Y1 2.5639068

5.2309 E-2 APY1 0.14219251

0.18275376 Y1P 0.27263657

0.15146312 Y2 6.1580 E-2

3.3672 E-2 APY2 0.12355565

0.44331725 Y2P 0.89273131

6.1580 E-2 AY1 0.15146312

4.7225921 AY2 4.2731781

Eigenvalues for Steady State No. 1

¯¯¯¯¯¯¯¯¯¯¯¯¯¯¯¯¯¯¯¯¯¯¯¯¯¯¯¯¯¯¯¯¯¯

-5878.2169

-4452.4167

-2510.4006

-1299.6516

-392.65837

-145.57513

1.7915605

Steady State No. 1 is unstable.

Eigenvalues for Steady State No. 2

¯¯¯¯¯¯¯¯¯¯¯¯¯¯¯¯¯¯¯¯¯¯¯¯¯¯¯¯¯¯¯¯¯¯

-6346.8262

-4927.26

-2866.2672

-1424.5087

-4.6933204

-355.14896

-189.03784

Steady State No. 2 is asymptotically stable.
